# Urinary bisphenol A and its substitutes exposure increased the risk of renal tubular injury (N-acetyl-*β*-d-glucosaminidase) in the general Taiwanese population

**DOI:** 10.3389/fpubh.2025.1505578

**Published:** 2025-05-19

**Authors:** Yu-Jung Lin, Jung-Wei Chang, Vinoth Kumar Ponnusamy, Han-Bin Huang, Hsin-Chang Chen, Po-Chin Huang

**Affiliations:** ^1^Intelligent Data Mining Laboratory, Department of Medical Research, Taichung Veterans General Hospital, Taichung, Taiwan; ^2^Institute of Environmental and Occupational Health Sciences, School of Medicine, National Yang Ming Chiao Tung University, Taipei, Taiwan; ^3^Department of Medicinal and Applied Chemistry, Kaohsiung Medical University, Kaohsiung City, Taiwan; ^4^Research Center for Precision Environmental Medicine, Kaohsiung Medical University, Kaohsiung City, Taiwan; ^5^School of Public Health, National Defense Medical Center, Taipei, Taiwan; ^6^Department of Chemistry, Tunghai University, Taichung, Taiwan; ^7^National Institute of Environmental Health Sciences, National Health Research Institutes, Miaoli, Taiwan; ^8^Department of Medical Research, China Medical University Hospital, China Medical University, Taichung, Taiwan; ^9^Department of Safety, Health and Environmental Engineering, National United University, Miaoli, Taiwan

**Keywords:** bisphenol A substitutes, hazard index, cumulative risk assessment, renal damage, Taiwan, bisphenol A

## Abstract

**Background:**

It is uncertain if exposure to BPA and its substitutes has an impact on renal function, including N-acetyl-β-D-glucosaminidase (NAG), which is an early marker for kidney injury. We aimed to (1) Estimate the daily intakes (DIs) of BPA and its substitutes using individual urinary levels and conduct the cumulative risk assessment of bisphenols. (2) Assessed the association between exposure to BPA and its substitutes with various renal function indices using a dose-based and cumulative risk assessment approach.

**Methods:**

We recruited 366 participants, and three bisphenols (BPA, bisphenol F, and bisphenol S) were analyzed through ultraperformance liquid chromatography–tandem mass spectrometry. DI levels were calculated for each bisphenol. Hazard index (HI) values were calculated for determining cumulative risk. Using the renal function index, we measured the serum and urinary level (e.g., microalbumin, NAG). The NAG/Creatinine ratio (> 4 IU/g creatinine) and other renal functions indexes based on clinical cut-off points to defined abnormality.

**Results:**

After adjustment for covariates, increased NAG/Creatinine ratios were associated with higher DIs of BPA, showing a dose-response trend (Adjusted Odds Ratio [AOR] _tertile2_: 3.58, 95% CI = 1.52–8.44; AOR _tertile3_: 7.34, 95% CI = 2.26–23.81; *P*_trend_ < 0.001). Notably, the HI of bisphenols was positively associated with NAG/Creatinine in adults (AOR _tertile2_= 2.18, 95% CI = 1.10–4.34; AOR _tertile3_= 4.27, 95% CI = 2.14–8.51) after adjusted for covariates.

**Conclusion:**

We found a sensitive risk factor for abnormal NAG/creatinine levels after exposure to BPA and its substitute. Further mechanistic studies are needed to clarify these associations.

## 1 Introduction

Chronic kidney disease (CKD) is a significant global health issue contributing to increased morbidity (1). Bisphenol A (BPA), an endocrine-disrupting chemical widely used in polycarbonate plastics and epoxy resins (2), applied in products such as thermal paper, toys, tableware, medical devices, polycarbonate bottles, food packaging, and cosmetic and personal care products (PCPs) (54). Notably, BPA concentrations in Taiwanese adults were nearly six times higher than in other countries, with levels of 7.96 μg/L in Taiwan, compared to 1.24 μg/L in the USA and 1.49 μg/L in Korea (3). BPA plays a crucial role in CKD progression as it is eliminated through the kidneys (4, 5).

Exposure to BPA triggers inflammation, leading to kidney injury, glomerular damage, tubular cell harm, and fibrosis. These nephrotoxicants enter cells via endocytosis, causing lipid peroxidation, mitochondrial dysfunction, DNA damage, and increased reactive oxygen species. This results in mutations, impaired cellular proliferation, and protein alterations, ultimately leading to renal damage (55). BPA adversely affects albumin-to-creatinine ratio (ACR) and estimated glomerular filtration rate (eGFR) (6), as confirmed by meta-analyses (6, 7). Despite restrictions on BPA use in many countries, substitutes like bisphenol F (BPF) and bisphenol S (BPS) have emerged, raising concerns due to their structural similarity and widespread application (8). Studies indicate BPS and BPF may pose toxicity risks comparable to or greater than BPA (9, 10), yet research on their impact on renal function remains limited (11, 12). Understanding the relationship between these substitutes and renal function is crucial.

Most human population studies have focused on the ACR (13) or on estimating functional parameters (e.g., eGFR) as outcomes of kidney diseases (7). However, the results for eGFR vary depending on the equations applied [e.g., Chronic Kidney Disease Epidemiology Collaboration [CKD-EPI] and Modification of Diet in Renal Disease [MDRD-4] equations (11)]. Although urinary N-acetyl-*β*-glucosaminidase (NAG) is crucial for estimating tubular injury (14, 15) and it has been identified as a sensitive determinant of CKD (16) and type 2 diabetes mellitus [DM (17)], whether exposure to BPA and its substitutes is associated urinary NAG levels remains unclear.

Given the limited literature on how exposure to BPA and its substitutes affects various renal indices in the general population, the present study aim to estimate the daily intakes of BPA and its substitutes using individual urinary levels and conduct the cumulative risk assessment of bisphenols. Using a cumulative risk assessment approach to assess the association between exposure to BPA and its substitutes with various renal function indices (e.g., ACR, eGFR, and NAG).

## 2 Methods

### 2.1 Study population

Participants were recruited from the Taiwan Environmental Survey for Toxicants 2013 (18–22) in the present cross-sectional study. The final study population comprised 271 adults (≥18 years) and 95 minors (<18 years).

### 2.2 Analytical method for detecting bisphenol

The analytical method used in the present study is described in detail in our previous study (19, 23). After >8 h of fasting, spot urine samples were also collected from the participants in the early morning during their visit. These samples were temporarily stored in polypropylene containers and subsequently transferred to amber glass bottles (prewashed with acetonitrile) and stored at −80°C until analysis.

### 2.3 DI estimation and cumulative risk assessment of BPA and its substitutes

The DI and cumulative risk assessment for BPA and its substitutes utilized methodologies detailed in our previous publications (3) (Specific calculation formulas are provided in the Supplementary Table S1).

### 2.4 Measurement of renal function and other parameters in serum and urine

We assessed various parameters related to renal function, including those obtained from urine and serum samples. The serum levels of blood creatinine, blood uric acid, and blood urea nitrogen (BUN) were determined. Additionally, concentrations of creatinine, microalbumin, protein, NAG, and uric acid in urine samples were measured. Upon collection in the morning, each participant’s blood and urine samples underwent centrifugation for 20 min at 4°C and were then stored at −80°C until analysis. All analyses were conducted in a blinded and randomized manner by a technician from a laboratory accredited by the Taiwan Accreditation Foundation (No. 1673) (18).

The ACR was determined by dividing the microalbumin level by the urinary creatinine concentration. The NAG-to-creatinine ratio (NAG/creatinine) was calculated by dividing the urine NAG level by the urinary creatinine concentration. eGFR was calculated using CKD-MDRD equation (24, 25) and the CKD-EPI equation (25, 26). Estimated creatinine clearance rate (CCr) was calculated using the Cockcroft–Gault formula (Supplementary Information: The formula of related renal functions, Supplementary Table S1).

Based on the Centers for Disease Control and Prevention (27), participants with fasting plasma glucose levels above 126 mg/dL were considered to have type 2 DM. According to the CKD Guideline (28) and the recommended clinical cut-off points for microalbumin (1.9 mg/dL), urine protein (14 mg/dL), ACR (30 mg/g creatinine), BUN (20 mg/dL), NAG/creatinine (4 IU/g creatinine) (29), and eGFR (90 mL/min/1.73 m^2^). Participants were also stratified into normal or abnormal groups indicative of early renal impairment (30).

### 2.5 Statistical analysis

BPA levels and renal function indicators were compared across different age groups using Mann–Whitney *U* or Kruskal-Wallis tests. The natural logarithms for urinary bisphenol levels and renal function indices were used to ensure that the normality assumption was met. The detectable rate was determined by dividing the number of urine samples in which the bisphenol level exceeded the detection limit by the total number of urine samples analyzed. The summary metric for BPs (ΣBPs) was calculated by summing the molar concentrations of the measured BPs (31). All bisphenols measurements, including the molar sum, were divided by urinary creatinine to adjust for urine dilution.

Urinary bisphenol levels, including DI, were categorized into tertiles (0–2), and p-trend tests were conducted to assess dose–response relationships with renal function indices. Covariates were selected on the basis of literature findings, their data availability, and their statistical significance in our models. After adjustment, we used multiple linear regressions and logistic regression models to investigate the associations between bisphenol levels and renal function indices in various models. All statistical analyses were performed using SAS (version 9.4; SAS Institute, Cary, NC, USA). A *p* value of <0.05 was regarded as significant.

Additionally, through the use of the mgcv R-package, log-transformed parameters were incorporated into generalized additive model (GAM)-penalized regression splines to determine the nonlinear associations with the risk pertaining to renal function indices. The optimal number of knots and the smoothing parameter were selected efficiently by conducting generalized cross-validation (32).

## 3 Results

### 3.1 Population characteristics

Table 1 summarizes the general and sociodemographic characteristics of the participants. The distribution of sexes was relatively even, with 128 men (47.2%) and 143 women (52.8%) in the adult group, and 55 boys (57.9%) and 40 girls (42.1%) in the minor group. Analysis revealed that males had a significantly higher frequency of abnormalities in urine protein levels (>14 mg/dL) and eGFR (<90 mL/min/1.73 m^2^) compared to females (urine protein: 11.7% vs. 2.1%, *p* = 0.002; eGFR: 50.0% vs. 37.1%, *p* = 0.035). Conversely, abnormalities in NAG/creatinine were more prevalent in females than males (44.4% vs. 35.4%). In contrast, abnormalities in renal function indices among minors were extremely rare (e.g., abnormal eGFR: 0%), and no significant sex differences were observed. Therefore, the investigation into the association between urinary bisphenol levels and renal function indices was limited to adults (Supplementary Table S2).

**Table 1:** Characteristics of the study population (*N* = 366).

Characteristics	Item	Children/Adolescents (<18 years, *N* = 95)		Adults (≥18 years, *n* = 271)	
		*n*	%	*n*	%
Gender	Girl/Female	40	42.1	143	52.8
	Boy/Male	55	57.9	128	47.2
Age (years)	7–12/18–40	49	51.6	64	23.6
	12–18/40–65	46	48.4	127	46.9
	65 and older	-	-	80	29.5
Region	Northern Taiwan	31	32.6	84	31.0
	Central Taiwan	15	15.8	37	13.7
	Southern Taiwan	22	23.2	77	28.4
	Eastern Taiwan	12	12.6	46	17.0
	Remote island	15	15.8	27	10.0
Marriage status	Single	94	99.0	46	17.0
	Married	1	1.0	197	72.7
	Divorce/widowed	0	0	28	10.3
Education	≦Elementary school	49	51.6	74	27.3
	Junior high school	29	30.5	39	14.4
	Senior high school	17	17.9	63	23.2
	≧College/graduates	0	0	95	35.1
Annual family income (USD)^a^	Below 15,625	37	42.1	151	58.1
	More than 15,625	51	57.9	109	41.9
Cigarette smoking^b^	Yes/No	2/93	2.1/97.9	65/205	24.1/75.9
Passive smoker^c^	Yes/No	49/45	52.1/47.9	135/135	50.0/50.0
Incense sticks^d^	Yes/No	29/66	30.5/69.5	147/123	54.4/45.6
PCPs usage^e^	Yes/No	83/11	88.3/11.7	197/69	74.1/25.9
Alcohol consumption^f^	Yes/No	1/93	1.1/98.9	35/232	13.1/86.9
Tea drinking^g^	Yes/No	46/49	48.4/51.6	156/114	57.8/42.2
Coffee drinking^h^	Yes/No	6/89	6.3/93.7	114/157	42.1/57.9
Betel nut chewing^i^	Yes/No	1/94	1.1/98.9	18/253	6.6/93.4
Pesticide use at home^j^	Yes/No	26/69	27.4/72.6	65/206	24.0/76.0

a The currency exchange rate of converting USD to new Taiwan dollar is 1:32.

b Subjects who self-reported consuming at least one cigarette per day.

c Subject who self-reported as lifelong nonsmokers (never-smokers) but involuntary inhalation of smoke from cigarettes or other tobacco.

d Subject who self-reported as having burnt incense at home ≥ weekly basis over the past 5 years.

e Subject who self-reported using at least one kind of PCPs, including body wash, lotion, perfume, and nail polishes.

f Subject consuming at least one bottle of alcohol drink per week.

g Subjects consuming at least one cup of tea or coffee per week.

h Subject chewing at least one betel nut per week.

i Subject chewing at least one betel nut per week.

j Subject who self-reported using household pesticides to control pests.

### 3.2 Distribution of urinary bisphenol levels and renal function index

The detection rate for BPA and its substitutes was 100% in all urine samples. Notably, the median levels for BPA and its substitutes were significantly higher in the adults than in the minors (BPA, 9.45 vs. 4.08 μg/g creatinine; BPF, 9.63 vs. 6.63 μg/g creatinine; BPS, 2.43 vs. 1.67 μg/g creatinine; ΣBPs, 0.10 vs. 0.06 nmol/g creatinine; all *p* < 0.001). We also performed comprehensive estimations of DI levels for BPA, BPF, and BPS. In the adults, the median DI level was 2.29 ng/kg/day for BPA, 2.35 ng/kg/day for BPF, and 0.58 ng/kg/day for BPS. These levels were significantly higher than those in the minors (*p* < 0.001), with median DI levels of 0.60 ng/kg/day for BPA, 0.77 ng/kg/day for BPF, and 0.24 ng/mL for BPS. Furthermore, the median HI values for BPA and its substitutes were significantly higher in the adults than in the minors (1.29 × 10^−3^ vs. 4.1× 10^−4^, *p* < 0.001; Table 2).

**Table 2:** Median and geometric mean levels of bisphenols and renal function index among Taiwanese adults and children/adolescents.

Variables	Children/Adolescents (<18 years, *N* = 95)			Adults (≥18 years, *N* = 271)			*p* ^b^
	<LOD ^a^	GM	Median	<LOD ^a^	GM	Median	
			(Interquartile range)			(Interquartile range)	
Bisphenols (μg/g creatinine) BPA	0	4.17	4.08 (2.68, 11.27)	0	9.79	9.45 (5.73, 18.26)	<0.001
BPF	0	6.85	6.63 (3.60, 12.04)	0	9.87	9.63 (5.58, 17.84)	<0.001
BPS	0	1.73	1.67 (1.00, 3.18)	0	2.36	2.43 (1.34, 4.35)	<0.001
ΣBPs (nmol/g creatinine)		0.06	0.06 (0.03, 0.11)		0.11	0.10 (0.06, 0.20)	<0.001
Daily intake of bisphenols (ng/kg/day)							
BPA		0.57	0.60 (0.33, 0.99)		2.35	2.29 (1.26, 4.24)	<0.001
BPF		0.94	0.77 (0.48, 1.82)		2.37	2.35 (1.30, 4.36)	<0.001
BPS		0.24	0.24 (0.14, 0.34)		0.57	0.58 (0.34, 1.05)	<0.001
HIRenal function factor		4.68 × 10^−4^	4.1 × 10^−4^ (2.55 × 10^−4^, 8.77 × 10^−3^)		1.35 × 10^−3^	1.29 × 10^−3^ (8.13 × 10^−4^, 2.45 × 10^−3^)	<0.001
Blood ^c^							
BUN (mg/dL)		10.31	10.30 (8.50, 12.50)		13.17	13.10 (10.10, 16.30)	<0.001
Creatinine (mg/dL)		0.62	0.62 (0.51, 0.74)		0.80	0.79 (0.66, 0.96)	<0.001
Uric acid (mg/dL)		5.65	5.65 (5.00, 6.80)		5.85	6.00 (4.80, 7.00)	0.311
eGFR (mL/min/1.73m^2^)		172.53	166.48 (128.60, 213.03)		93.31	94.50 (82.22, 110.06)	<0.001
CCr (mL/min)		136.13	132.21 (108.42, 166.85)		101.61	104.34 (79.66, 132.89)	<0.001
Urine ^d^							
Creatinine (mg/dL)		102.52	99.00 (70.10, 148.21)		78.92	81.00 (48.00, 122.00)	0.001
Microalbumin (mg/dL)		0.37	0.25 (0.25, 0.50)		0.43	0.25 (0.25, 0.64)	0.343
pH		6.16	6.50 (6.00, 6.50)		6.03	6.50 (5.00, 6.50)	0.130
Protein (mg/dL)		5.66	5.50 (3.40, 8.80)		3.59	3.80 (2.30, 6.50)	<0.001
Uric acid (mg/dL)		40.17	42.20 (24.20, 68.10)		32.03	32.80 (22.20, 50.60)	0.003
NAG (IU/L) ^e^		1.47	1.58 (0.79, 3.27)		2.63	2.63 (1.64, 4.49)	<0.001
ACR (mg/g)		3.60	3.21 (2.07, 5.10)		5.40	4.55 (2.78, 7.44)	0.001
NAG/creatinine (IU/g) ^e^		1.42	1.59 (1.01, 2.45)		3.32	3.35 (2.00, 5.88)	<0.001

GM, geometric mean; LOD: limit of detection; *p*, *p*-value; ΣBPs: the bisphenol weighted molar sum; BUN, blood urea nitrogen; eGFR, Estimated glomerular filtration rate based on CKD- MDRD equation; CCr, creatinine clearance; NAG, N-acetyl-beta-D-glucosaminidase; ACR, microalbumin-to-creatinine ratio; NAG/creatinine, NAG-to-creatinine ratio.

a The limits of detection (LOD) for BPA, BPF and BPS were 0.08, 0.07 and 0.10, respectively.

b Mann–Whitney *U* test calculated for the difference in means between adults and minors.

c Sample size of children and adolescent population = 74, sample size of adult population = 266.

d Sample size of children and adolescent population = 95, Sample size of adult population = 271.

e Sample size of children and adolescent population = 92, Sample size of adult population = 269.

Relative to the minors, the adults exhibited significantly higher median levels of blood BUN (13.10 vs. 10.30 mg/dL), blood creatinine (0.79 vs. 0.62 mg/dL), urine NAG (2.63 vs. 1.58 IU/L), ACR (4.55 vs. 3.21 mg/g), and NAG/creatinine (3.35 vs. 1.59 IU/g). By contrast, the median levels of urine creatinine (81 vs. 99 mg/dL), urine protein (3.8 vs. 5.5 mg/dL), uric acid (32.8 vs. 42.2 mg/dL), eGFR (94.50 vs. 166.48 mL/min/1.73 m^2^), and CCr (104.34 vs. 132.21 mL/min) were significantly lower in the adults than in the minors (Table 2).

### 3.3 Association between bisphenol levels and renal function index

The DI levels for BPA and its substitutes were significantly and positively associated with the ACR and NAG/creatinine levels (ACR, [BPA, *r* = 0.25; BPF, *r* = 0.26; BPS, *r* = 0.26]; NAG/creatinine, [BPA, *r* = 0.42; BPF, *r* = 0.38; BPS, *r* = 0.35]; all *p* < 0.05). Additionally, the DI levels for BPA and its substitutes were significantly and negatively associated with urine protein levels and eGFR (urine protein, [BPA, *r* = −0.39; BPF, *r* = −0.42; BPS, *r* = −0.42]; eGFR, [BPA, *r* = −0.33; BPF, *r* = −0.20; BPS, *r* = −0.22]; all *p* < 0.05) (Supplementary Figure S1).

Supplementary Tables S3, S4 reveal that higher HI values were linked to lower urine protein (*β* = −0.66, *p* < 0.001) and NAG (*β* = −0.25, *p* < 0.001) levels, but higher ACR (*β* = 0.39, *p* < 0.001) and NAG/creatinine (*β* = 0.32, *p* < 0.001) levels. Overall, while exposure to BPA and its substitutes appears to mildly affect renal function, no association with eGFR was observed.

**Figure 1:**
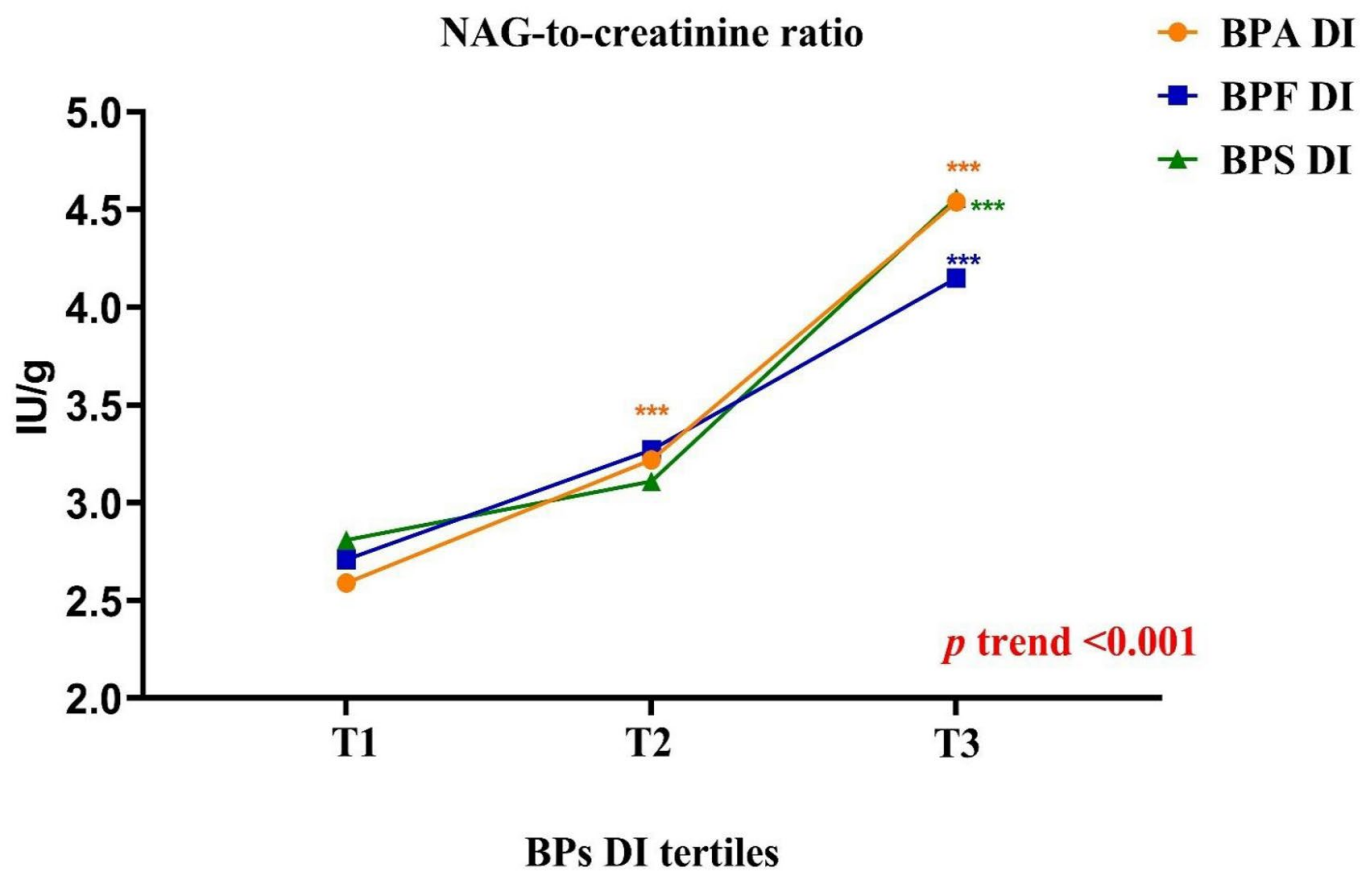
Schematic representation of the results obtained in the parameters related to renal function according to the daily intake of bisphenols tertiles in adults (BPA: [T1 < 1.11; T2 = 1.11–2.83; T3 > 2.83]; BPF: [T1 < 1.24; T2 = 1.24–2.80; T3 > 2.80]; BPS: [T1 < 0.41; T2 = 0.41–0.81; T3 > 0.81]). All results were expressed as median. Kruskal–Wallis test followed by Dunn’s multiple comparisons test. ^*^, *p* < 0.05; ^**^, *p* < 0.01; ^***^, *p* < 0.001.

Higher bisphenol DI levels were associated with lower NAG levels (p-trend <0.001), but higher NAG/creatinine ratios (p-trend <0.001) among adults. Specifically, adults in the highest DI tertile exhibited notably elevated NAG/creatinine compared to those in the lowest tertile (Supplementary Tables S5, S6, Figure 1). These findings indicate that higher bisphenol exposure levels were linked to increased abnormalities in renal function indices, particularly NAG/creatinine, in adults.

**Figure 2:**
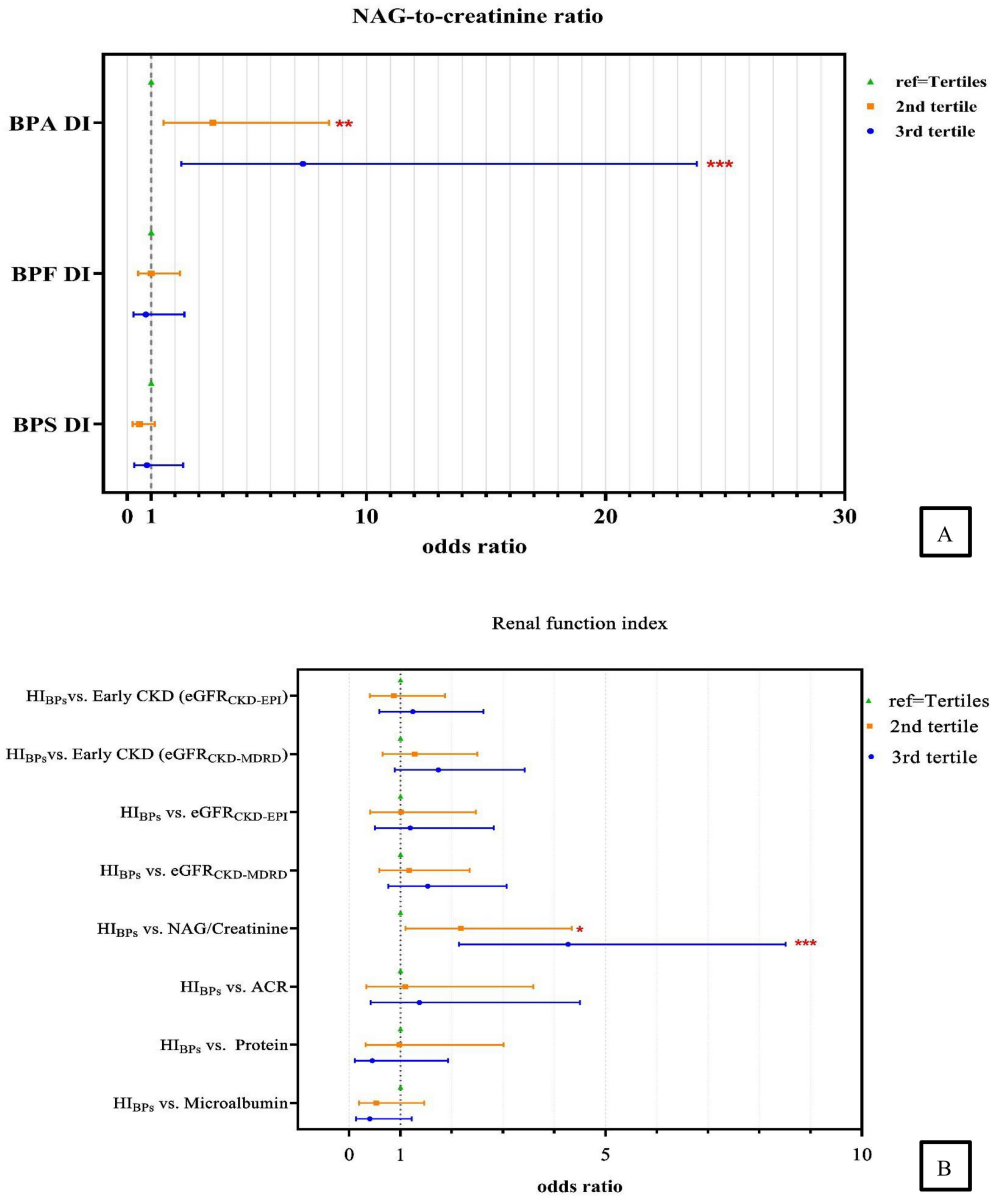
Association between **(A)** urinary measurement of daily bisphenols intake and the risk of higher NAG-to-creatinine ratio; **(B)** the HI and renal function indexes. Abnormality: Microalbumin>1.9 mg/dL; Urine protein >14 mg/L; Albumin-to-creatinine ratio (ACR) >30 mg/g; N-acetyl-*β*-D-glucosaminidase to creatinine ratio (NAG/Creatinine) > 4 IU/g; Estimated glomerular filtration rate (eGFR_CKD-MDRD/EPI_) < 90 mL/min/1.73 m^2^ and eGFR based on CKD- MDRD, CKD-EPI equation, respectively; Early Chronic Kidney Disease (Early CKD_CKD-MDRD/EPI_): 60 ≤ eGFR <90 mL/min/1.73 m^2^ and eGFR based on CKD- MDRD and CKD-EPI equation, respectively; Adjustment of age, sex, type 2 DM, and BMI. ^*^, *p* < 0.05; ^**^, *p* < 0.01; ^***^, *p* < 0.001.

After adjustments for type 2 DM, BMI age and sex, we discovered that the adjusted odds ratio (AOR) for the adults in the highest tertile (T3) of BPA DI had a 7.34 times higher risk (AOR = 7.34, 95% confidence interval [CI] = 2.26–23.81) of having abnormal NAG/creatinine compared with those in the lowest tertile. Furthermore, those in the second tertile (T2) had a 3.58 times higher risk (AOR = 3.58, 95% CI = 1.52–8.44; Table 3, Figure 2) of having abnormal NAG/creatinine compared with those in the lowest tertile. No similar trend was identified for the other renal function indices. This finding indicates that the risk of having abnormal NAG/creatinine increased by 4 to 7 times with BPA DI, with a dose–response relationship. Although, the third tertile of urinary BPS and BPS DI levels associated with abnormality urine proteins (BPS: AOR = 12.23, 95% CI = 1.61–93.16; BPS DI: AOR = 7.24, 95% CI = 1.00–52.24), however, the 95% CI was rather wide and existed uncertainty relationships, because there was so scanty data on the abnormality protein (*n* = 18). We also discovered that urinary BPS level in the higher tertile (tertile 2 and 3) of BPS DI had a 5.53 to 5.98 times higher risk of abnormal ACR compared with those in the lowest tertile (*p* < 0.05).

**Table 3:** Association between bisphenols levels and the risk of higher renal function indexes in adults (*n* = 271).

Analyte	Microalbumin ^a^			Protein ^b^			ACR ^c^		
	Case/ *N* (%)	AOR (95% CI)	*p*	Case/ *N* (%)	AOR (95% CI)	*p*	Case/ *N* (%)	AOR (95% CI)	*p*
Model 1 ^d^									
BPA									
<6.38	12/90 (13.3)	1	-	5/90 (5.6)	1	-	9/90 (10)	1	-
6.38–10.12	7/91 (7.7)	0.67 (0.21, 2.13)	0.496	5/91 (5.8)	0.79 (0.17, 3.72)	0.768	5/91 (5.5)	0.67 (0.17, 2.60)	0.566
>10.12	9/90 (10)	0.54 (0.14, 2.13)	0.379	8/90 (9.5)	0.69 (0.12, 3.94)	0.675	8/90 (8.9)	0.49 (0.11, 2.09)	0.334
BPF									
<6.13	10/90 (11.1)	1	-	6/90 (6.7)	1	-	7/90 (7.8)	1	-
6.13–10.13	9/91 (9.9)	0.73 (0.22, 2.39)	0.602	6/91 (7.1)	0.35 (0.07, 1.64)	0.181	7/91 (7.7)	0.61 (0.15, 2.43)	0.480
>10.13	9/90 (10)	1.03 (0.27, 3.85)	0.969	6/90 (7.1)	0.36 (0.07, 1.88)	0.225	8/90 (8.9)	0.62 (0.14, 2.70)	0.523
BPS									
<1.58	9/91 (9.9)	1	-	3/91 (3.4)	1	-	5/91 (5.5)	1	-
1.58–2.48	10/91 (11)	1.48 (0.44, 4.99)	0.528	5/91 (5.6)	2.14 (0.37, 12.22)	0.393	9/91 (9.9)	5.53 (1.16, 26.44)	0.032^*^
>2.48	9/89 (10.1)	1.94 (0.46, 8.25)	0.368	10/89 (12.4)	12.23 (1.61, 93.16)	0.016^*^	8/89 (9)	5.98 (1.04, 34.21)	0.045^*^
Model 2 ^e^									
BPA DI									
<1.64	16/90 (17.8)	1	-	9/90 (10)	1	-	9/90 (10)	1	-
1.64–3.61	6/91 (6.6)	0.32 (0.09, 1.19)	0.089	6/91 (6.7)	0.44 (0.11, 1.86)	0.266	5/91 (5.5)	0.29 (0.06, 1.39)	0.121
>3.61	6/90 (6.7)	0.24 (0.04, 1.47)	0.122	3/90 (3.8)	0.17 (0.02, 1.49)	0.109	8/90 (8.9)	0.22 (0.03, 1.53)	0.126
BPF DI									
<1.61	13/90 (14.4)	1	-	7/90 (7.8)	1	-	8/90 (8.9)	1	-
1.61–3.46	9/91 (9.9)	0.98 (0.30, 3.18)	0.970	8/91 (9)	1.36 (0.35, 5.25)	0.652	6/91 (6.6)	0.58 (0.15, 2.28)	0.439
>3.46	6/90 (6.7)	0.63 (0.09, 4.58)	0.648	3/90 (3.8)	0.37 (0.04, 3.63)	0.396	8/90 (8.9)	0.55 (0.07, 4.17)	0.559
BPS DI									
<0.41	14/90 (15.6)	1	-	7/90 (7.8)	1	-	5/90 (5.6)	1	-
0.41–0.81	6/91 (6.6)	0.81 (0.23, 2.87)	0.744	5/91 (5.6)	1.30 (0.30, 5.53)	0.727	7/91 (7.7)	4.28 (0.91, 20.19)	0.066^†^
>0.81	8/90 (8.9)	2.37 (0.36, 15.56)	0.370	6/90 (7.5)	7.24 (1.00, 52.24)	0.050^*^	10/90 (11.1)	14.40 (1.52, 136.46)	0.020^*^
Model 3 ^e^									
HI ^e^									
<0.001	14/90 (15.6)	1	-	8/90 (8.9)	1	-	7/90 (7.8)	1	-
0.001–0.002	8/91 (8.8)	0.53 (0.19, 1.46)	0.218	7/91 (7.9)	0.98 (0.32, 3.01)	0.965	7/91 (7.7)	1.09 (0.33, 3.59)	0.892
>0002	6/90 (6.7)	0.40 (0.13, 1.22)	0.107	3/90 (3.8)	0.45 (0.11, 1.93)	0.284	8/90 (8.9)	1.37 (0.42, 4.50)	0.600
AOR, Adjusted Odds ratio; ACR, microalbumin-to-creatinine ratio; *p*, *p*-value; ^a^ Microalbumin > 1.9 mg/dL; ^b^ Urine protein > 14 mg/L; ^c^ ACR > 30 mg/g; ^d^ Adjustment of age, sex, type 2 DM, urine creatinine and BMI; ^e^ Adjustment of age, sex, type 2 DM, and BMI; ^†^, *p* < 0.1;^*^, *p* < 0.05; ^**^, *p* < 0.01; ^***^, *p* < 0.001.									

Analyte	NAG/Creatinine ^a^			eGFR ^b^			Early chronic kidney disease ^c^		
	Case/ *N* (%)	AOR (95% CI)	*p*	Case/ *N* (%)	AOR (95% CI)	*p*	Case/ *N* (%)	AOR (95% CI)	*p*
Model 1 ^d^									
BPA									
<6.38	32/89 (36)	1	-	33/88 (37.5)	1	-	25/88 (28.4)	1	-
6.38–10.12	35/91 (38.5)	1.49 (0.71, 3.10)	0.288	40/90 (44.4)	1.91 (0.90, 4.07)	0.092	37/90 (41.1)	2.59 (1.23, 5.45)	0.012^**^
>10.12	41/89 (46.1)	1.92 (0.83, 4.43)	0.128	42/88 (47.7)	1.66 (0.70, 3.94)	0.252	39/88 (44.3)	2.63 (1.12, 6.15)	0.026^*^
BPF									
<6.13	35/90 (38.9)	1	-	35/87 (40.2)	1	-	30/87 (34.5)	1	-
6.13–10.13	35/90 (38.9)	0.92 (0.44, 1.91)	0.812	38/90 (42.2)	1.01 (0.48, 2.14)	0.971	32/90 (35.6)	0.99 (0.48, 2.07)	0.985
>10.13	38/89 (42.7)	0.85 (0.38, 1.92)	0.701	42/89 (47.2)	1.21 (0.52, 2.80)	0.658	39/89 (43.8)	1.36 (0.60, 3.06)	0.459
BPS									
<1.58	37/90 (41.1)	1	-	37/88 (41.6)	1	-	34/89 (38.2)	1	-
1.58–2.48	38/91 (41.8)	1.08 (0.52, 2.25)	0.828	38/89 (42.7)	0.82 (0.39, 1.74)	0.611	32/89 (36)	0.59 (0.28, 1.24)	0.162
>2.48	33/88 (37.5)	0.66 (0.29, 1.52)	0.328	40/88 (45.5)	0.98 (0.42, 2.32)	0.968	35/88 (39.8)	0.62 (0.27, 1.42)	0.255
Model 2 ^e^									
BPA DI									
<1.64	19/89 (21.1)	1	-	32/88 (36.4)	1	-	27/88 (30.7)	1	-
1.64–3.61	36/91 (40)	3.58 (1.52, 8.44)	0.004^**^	41/90 (45.6)	1.57 (0.68, 3.59)	0.290	35/90 (38.9)	1.58 (0.71, 3.52)	0.260
>3.61	53/89 (59.6)	7.34 (2.26, 23.81)	0.001^***^	42/88 (47.7)	0.97 (0.30, 3.12)	0.960	39/88 (44.3)	1.55 (0.51, 4.71)	0.443
BPF DI									
<1.61	25/90 (27.8)	1	-	36/89 (40.5)	1	-	30/89 (33.7)	1	-
1.61–3.46	35/91 (38.5)	0.99 (0.45, 2.20)	0.981	38/89 (42.7)	0.88 (0.39, 1.98)	0.751	34/89 (38.2)	1.08 (0.50, 2.34)	0.848
>3.46	48/88 (54.6)	0.78 (0.26, 2.39)	0.666	41/88 (46.6)	0.73 (0.23, 2.27)	0.586	37/88 (42.1)	0.90 (0.30, 2.66)	0.845
BPS DI									
<0.41	28/90 (31.1)	1	-	33/89 (37.1)	1	-	30/89 (33.7)	1	-
0.41–0.81	29/89 (32.6)	0.51 (0.22, 1.15)	0.105	39/89 (43.8)	1.19 (0.53, 2.67)	0.679	33/89 (37.1)	0.85 (0.39, 1.84)	0.675
>0.81	51/90 (56.7)	0.82 (0.29, 2.33)	0.709	43/88 (48.9)	2.50 (0.82, 7.56)	0.106	38/88 (43.2)	1.31 (0.46, 3.69)	0.611
Model 3 ^e^									
HI ^e^									
<0.001	21/90 (23.3)	1	-	35/89 (39.3)	1	-	29/89 (32.6)	1	-
0.001–0.002	35/90 (28.9)	2.18 (1.10, 4.34)	0.027^*^	36/89 (40.5)	1.17 (0.59, 2.35)	0.653	32/89 (36)	1.28 (0.65, 2.50)	0.478
>0002	32/89 (58.4)	4.27 (2.14, 8.51)	<0.001^***^	44/88 (50)	1.53 (0.76, 3.07)	0.236	40/88 (45.5)	1.74 (0.89, 3.42)	0.108
AOR, Adjusted Odds ratio; *p*, *p*-value; eGFR, Estimated glomerular filtration rate; NAG/Creatinine, N-acetyl-β-D-glucosaminidase to creatinine ratio; ^a^ NAG/Creatinine > 4 IU/g; ^b^ eGFR < 90 mL/min/1.73 m^2^ and eGFR based on CKD- MDRD equation; ^c^ 60 ≤ eGFR < 90 mL/min/1.73 m^2^ and eGFR based on CKD- MDRD equation; ^d^ Adjustment of age, sex, type 2 DM, and BMI; ^†^, *p* < 0.1;^*^, *p* < 0.05; ^**^, *p* < 0.01; ^***^, *p* < 0.001.									

In the highest tertile of BPS DI (third tertile), the risk of having an abnormal eGFR _CKD-EPI_ was 4.06 times higher (AOR = 4.06, 95% CI = 0.97–17.07, *p* < 0.1) (Supplementary Table S7); this finding was inconsistent to that obtained using the eGFR_CKD-MDRD_, which is based on the CKD-MDRD equation, no significant differences were observed (AOR = 2.50, 95% CI = 0.82–7.56; Table 3). Furthermore, when early CKD was defined on the basis of abnormalities in eGFR (depend on CKD-MDRD equation), we found that urinary BPA levels increased the risk of early CKD by 2.50 times (AOR = 2.50, 95% CI =1.19–5.24, 2nd tertile) and 2.55 times (AOR = 2.55, 95% CI =1.09–5.95, 3rd tertile), but no significant differences were observed depend on CKD-EPI equation (Supplementary Table S7).

**Figure 3:**
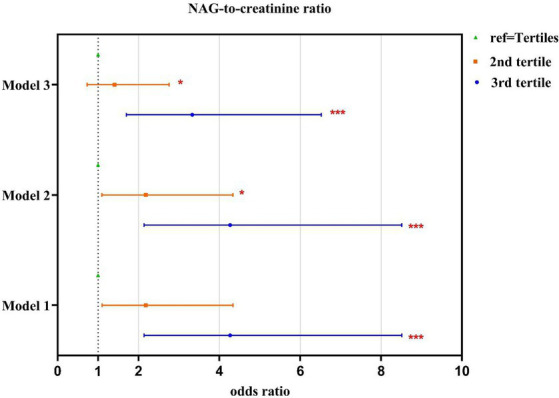
Association between the HI_BPs_ and NAG-to-creatinine ratio. HI is the cumulative summation of HQs for each compound; Model 1: based on EFSA (46) TDI of BPA (4,000 ng/kg/ay), TDI of 4,000 (ng/kg bw /day) for BPF (56); TDI of 4,400 (ng/kg bw /day) for BPS; Model 2: based on EFSA (47) TDI of BPA (0.2 ng/kg/day) and assume the BPA TDI equal to BPF and BPS; Model 3: based on BfR (48) TDI (200 ng/kg/day) and assume the BPA TDI equal to BPF and BPS; Adjustment of age, sex, type 2 DM and BMI in all models; ^*^, *p* < 0.05; ^**^, *p* < 0.01; ^***^, *p* < 0.001.

We found that excluding patients with DM or adjusting for this condition did not alter the strength of association between the risks of abnormal NAG/creatinine, whereas highest cumulative HI was significantly positively associated with abnormal NAG/creatinine (models 1–3) (Supplementary Figure S2). After adjustment for covariates, the AOR for adults in the highest tertile of HI value, compared to the lowest tertile group, showed a 4.27 times higher risk (95% CI = 2.14–8.51) of abnormal NAG/creatinine, followed by the second tertile (T2) with a 2.18 times higher risk (95% CI = 1.10–4.34), suggesting that the cumulative risk of bisphenol exposure increases the risk of renal tubular damage (Table 3, Figure 2). Our study also examined the relationship between the cumulative risk of HI calculated based on different tolerable daily intakes (TDIs) and the NAG/creatinine. The cumulative HI for bisphenols were significantly and positively associated with NAG/creatinine, especially the highest tertile of HI, the risk of having an abnormal NAG/creatinine were 3.33 to 4.27 times higher (Model 1: AOR = 4.27, 95% CI = 1.19–8.51; Model 2: AOR = 4.27, 95% CI = 2.14–8.51; Model 3: AOR = 3.33, 95% CI = 1.70–6.52) (Supplementary Table S8, Figure 3).

The GAM model and the penalty spline method were applied to determine any significant departure from linearity in this relationship (Supplementary Figure S3). The results revealed that BPA DI and HI values were associated abnormal NAG/creatinine (*P_smooth_* < 0.001). Furthermore, a similar trend was identified between the BPS concentration/DI and renal function indexes, i.e., abnormalities in the early CKD (depend on CKD-MDRD equation) (*P_smooth_* < 0.05).

## 4 Discussion

We identified a significant dose–response relationship between increasing BPA DI and the likelihood of elevated NAG/creatinine levels in Taiwanese adults aged 18 years and older. Notably, we also discovered that HI value was significantly and positively associated with NAG/creatinine after adjustment for significant covariates.

Studies have reported inconsistent findings regarding the associations between BPA exposure and renal function. Notably, most clinical markers of renal function are based on the ACR or eGFR. An increase in the urinary BPA concentration is significantly associated with an increase in the ACR (6, 33) and a decrease in eGFR (6). By contrast, a positive association between urinary BPA levels and eGFR was reported in female adults from the general population in the United States (5) and in patients with CKD (8). A possible explanation for this association is that declining renal function increases the difficulty of eliminating BPA, resulting in BPA accumulation and a vicious cycle of BPA accumulation, especially in individuals with an eGFR of 60 mL/min/1.73 m^2^ (34). In the U.S. National Health and Nutrition Examination Survey from 2003 to 2016, analysis of data from 12,000 adults based on BPA/creatinine quartiles showed significantly higher ACR in groups with higher urinary BPA levels compared to those with the lowest levels (7). A similar trend was revealed in the BPA substitutes and renal function; the results revealed that individuals with higher urinary BPS levels exhibited higher ACR and eGFR (11), whereas those with higher urinary BPF levels exhibited lower eGFR. These findings were similar to those of the present study; that is, urinary BPS levels are positively associated with an abnormal ACR. In contrast to the findings related to BPA, which showed a significant increase in urea nitrogen, serum creatinine, 24-h proteinuria, and the urine protein-to-creatinine ratio, as well as a significant reduction in creatinine clearance, our study revealed a different outcome. Higher BPS concentrations were associated with an increased risk of proteinuria abnormalities (7). However, few study has assessed the association between bisphenol DI and renal function. We confirmed a significant positive association between BPA exposure and the risk of having higher NAG/creatinine in adults. Furthermore, the highest BPS DI was associated with an increased risk of abnormalities in protein and eGFR by CKD-EPI equation. Due to the short half-life of bisphenols, relying solely on a single urinary marker may not adequately reflect renal function. Hence, we assessed both short-term (concentration and DI) and long-term (cumulative risk) exposures to bisphenol to comprehensively examine the relationships between bisphenol exposure and renal function indices.

Studies examining eGFR by applying various equations (e.g., CKD-EPI or MDRD-4) have obtained inconsistent results (26). Nevertheless, eGFR is a crucial basis for diagnosing CKD (28). In Taiwan, MDRD equations are used to calculate eGFR as part of efforts for the prevention and treatment of CKD (35), and numerous Taiwanese studies have applied the CKD-MDRD equation to calculate eGFR (7, 36). In the present study, we discovered that BPS DI levels were positively associated with abnormalities in eGFR when the CKD-EPI equation was applied; however, no significant differences in eGFR were identified when the CKD-MDRD equation was applied. Additionally, the urinary BPA levels increased the early CKD when early CKD (60 ≤ eGFR<90 mL/min/1.73 m^2^) depend on CKD-MDRD equation, whereas there was no significant difference between urinary BPA levels and early CKD depend on CKD-EPI equation. This finding is similar to that of Moreno-Gómez-Toledano et al. (7), who identified a significant difference between higher levels of urinary BPA and lowest level only when the CKD-EPI equation was applied (i.e., a significant difference was not identified when the CKD-MDRD equation was applied). Therefore, relying only on clinical indicators such as eGFR and ACR is insufficient as part of efforts for CKD prevention.

Urinary NAG is widely used as a valuable biomarker of both acute kidney injury and CKD (37). Furthermore, urinary NAG is a sensitive indicator of proximal tubular cell injury (38), and an increase in the urinary NAG concentration suggests injury to the proximal tubule. Given the fundamental roles of NAG, it is an essential contributor to chronic diseases such as diabetes (39), resulting in diabetic nephropathy (40). Notably, NAG levels are already elevated in patients with diabetes who have a normal albumin level and a normal eGFR (41). In the present study, after adjustment for covariates (including DM), high BPA DI increased a high risk of NAG/creatinine abnormalities, namely BPA still dominated bisphenol exposure in our study despite restrictions on its use and production (42), possibly because Taiwan banned only BPA used in baby bottles. Nevertheless, researchers are yet to explore the relationship between bisphenol exposure and abnormal NAG/creatinine levels; most research has focused only on the association between phthalate exposure patterns and renal impairment and has reported that high-molecular-weight phthalate pattern scores were positively associated with NAG levels in adults in Shanghai (43). Additionally, an increase in two ubiquitous chemicals (urinary melamine and estimated diethyl hexyl phthalate [DEHP] intake) together may be positively associated with an increase in urinary NAG/creatinine levels in pregnant women in Taiwan (44). This finding is similar to our findings; that is, bisphenols and phthalates exhibit similar properties, and both may cause renal tubular injury. In conclusion, BPA exposure does not appear to be related to glomerular filtration rate, with a significant association found only with its substitute BPS. This suggests that BPA exposure is more likely to cause renal tubular injury. Given the high BPA exposure in the Taiwanese population (3), it is crucial to pay close attention to the potential impact of kidney damage in the future.

Limited studies provide dose- or risk-based predictors for extrapolating the adverse renal effects of bisphenol exposure. Uncertainty in risk assessment arises from factors like exposure scenario characterization, parameter estimates, and model predictions (45). Variations in TDI based on different health effects for the same chemical substance lead to diverse hazard risk calculations. Therefore, we propose a reference range for uncertain risk scenarios. Based on European Food Safety Authority (EFSA)’s TDI for BPA, focused on renal toxicity (46), we utilized HI values to evaluate cumulative bisphenol exposure and its association with reduced renal function indices in Taiwanese adults, consistent with our previous approach (18). After adjusting for covariates such as DM, our analysis indicated that higher HI values were associated with increased risk of abnormal NAG/creatinine, aligning with EFSA’s TDI (47) and contrasting with The German Federal Institute for Risk Assessment, BfR’s TDI (48), which underestimated this risk. BPA is recognized as a biomarker of renal disease with nephrotoxic effects reported (46, 49, 50). However, studies on the nephrotoxicity of BPA substitutes at human exposure levels are sparse. For BPF and BPS, TDIs were calculated assuming their renal effects are similar to BPA (31, 51). More research on the nephrotoxic effects of various BPA substitutes is essential to mitigate uncertainty in risk assessment. As international BPA regulations vary and remain controversial, the EFSA CEP Panel identified BPA’s effect on Th17 cell percentage as the critical effect. After dose conversion to HED, the lowest BMDL of 8.2 ng/kg bw per day was used for risk assessment, with default UFs of 2.5 and 10 applied for inter-species toxicodynamic and intra-human variability (47). The BfR disagrees with the EFSA’s new TDI, citing methodological discrepancies, particularly the lack of evidence that increased Th17 cells in mice cause adverse effects (48). Despite this, we recommend that countries set region-specific BPA management or restriction guidelines based on local exposure situations and economic conditions. Additionally, more stringent regulations for BPA substitutes should be introduced step by step.

The present study has several limitations. Firstly, its cross-sectional design hinders establishing causality despite associations found between BPA and its substitutes with renal function indices. Secondly, potential alternative explanations for our results cannot be ruled out due to unmeasured confounding factors like phthalates, melamine, and other metals in our regression models (18, 51, 52). Thirdly, using single spot urine samples instead of 24-h collections may introduce bias in assessing associations of BPA and its substitutes with renal biomarkers, although some studies suggest single spot samples can indicate long-term exposure when exposure levels are stable (53). To mitigate this, we employed multiple exposure indicators (DI and HI value) and designed our questionnaire to assess exposure frequency assuming participants had similar lifestyles and dietary habits. Fourthly, using serum creatinine to adjust for renal function indices like eGFR or ACR may be limited by differences in serum creatinine levels based on individual characteristics such as age, sex, or race/ethnicity. Finally, the toxicity of BPA substitutes regarding human exposure and renal damage effects remains understudied, with TDIs for BPF and BPS assumed based on their similarity to BPA’s renal effects in HI evaluations from prior research. Prospective studies are needed to conclusively determine whether BPA and its substitutes are nephrotoxic.

Although our study population was smaller compared to other human biomonitoring datasets, we found significant positive associations between higher BPA DI and the cumulative risk of bisphenols with increased NAG/creatinine levels in adults. Elevated NAG/creatinine levels indicate a higher risk of renal tubular injury and early-stage kidney disease. Comprehensive or mechanistic studies are needed to further elucidate this association. Incorporating other renal function indicators such as NAG/creatinine as clinical diagnostic markers in future studies is recommended. Besides, we also suggest that the sample size of participants be continuously increased and future long-term follow-up studies be conducted to establish a clearer causal relationship.

## Data Availability

The original contributions presented in the study are included in the article/Supplementary material, further inquiries can be directed to the corresponding author.
