# Supplementary material for: Urinary bisphenol A and its substitutes exposure increased the risk of renal tubular injury (N-acetyl-β-d-glucosaminidase) in the general Taiwanese population
Source: Front Public Health. 2025 May 19;13:1505578. doi: 10.3389/fpubh.2025.1505578 (PMC12128855; doi:10.3389/fpubh.2025.1505578)
Supplement: Supplementary file 1 [file Data_Sheet_1.docx]

**Supplementary material**

Urinary Bisphenol A and its Substitute Exposure Increased the Risk of Renal Tubular Injury (N‐acetyl‐β‐d‐glucosaminidase) in the general Taiwanese population

Yu-Jung Lin^1^, Jung-Wei Chang^2^, Vinoth Kumar Ponnusamy^3,4^, Han-Bin Huang^5^, Hsin-Chang Chen^6^, Po-Chin Huang^1,4,7,8 *^

^1^ National Institute of Environmental Health Sciences, National Health Research Institutes, Miaoli, Taiwan

^2^ Institute of Environmental and Occupational Health Sciences, School of Medicine, National Yang Ming Chiao Tung University, Taipei, Taiwan,

^3^ Department of Medicinal and Applied Chemistry, Kaohsiung Medical University, Kaohsiung City, Taiwan, ^4^ Research Center for Precision Environmental Medicine, Kaohsiung Medical University, Kaohsiung City, Taiwan,

^5^ School of Public Health, National Defense Medical Center, Taipei, Taiwan

^6^ Department of Chemistry, Tunghai University, Taichung, Taiwan,

^7^ Department of Medical Research, China Medical University Hospital, China Medical University, Taichung, Taiwan

^8^ Department of Safety, Health and Environmental Engineering, National United University, Miaoli, Taiwan

**Corresponding Author (*)**:

Po-Chin Huang, PhD, Associate Investigator,

National Institute of Environmental Health Sciences,

National Health Research Institutes, 35 Keyan Road, Zhunan, Miaoli County, 35035,

Email: [pchuang@nhri.edu.tw](mailto:pchuang@nhri.edu.tw)

Supplementary Information

1. **Methods**
   1. *DI estimation and Cumulative risk assessment of BPA and its substitutes*

The negative effects of chronic bisphenol exposure on renal function were assessed through the analysis of data on urinary bisphenol levels with back-calculation.

$$DI of bisphenol [\frac{\left( \frac{\mu g}{kg} \right)}{day}]=\frac{UE \left( \frac{\mu g}{g} \right)\times CE (\frac{\frac{mg}{kg}}{day})}{F_{UE}\times BW \left( Kg \right)\times1000(\frac{mg}{g})}(Eq. 1)$$

In Eq. 1, UE = creatinine-adjusted bisphenol (micrograms per gram of creatinine), BW = body weight, and CE = daily creatinine excretion.

For cumulative risk assessment, we calculated hazard quotients (HQs) and HI values. HQs were calculated to quantitatively assess the potential health hazards of bisphenol (HQs were computed taking into account the tolerable daily intake (DI) and a reference dose, supplementary information). HI is the sum of the HQs for each type of bisphenol. The following formula was used to calculate HQs: $\text{HQ}_{\mathrm{TDI}}\text{ }\text{=}\text{ }\frac{\mathrm{DI}}{\mathrm{TDI}}\left( \text{Eq. 2} \right)$

HI values were calculated using the following formula: $HI = \Sigma HQ\left( \text{Eq. 3} \right)$

On the basis of the kidney toxicity effects of BPA, the European Food Safety Authority (EFSA, 2015) recommends a tolerable DI (TDI) of 4,000 ng/kg bw /day. Lin et al. (2022) assume the BPF toxicity equal to BPA, recommended a TDI of 4,000 (ng/kg bw /day) for BPF, and Mok et al. (2021) converted by mole recommended a TDI of 4,400 (ng/kg bw /day) for BPS. Additionally, EFSA (2023) re-established a new TDI of 0.2 ng/kg bw /day for BPA based on critical endpoints of T helper cell, which is 20,000 times lower than the previous TDI of 4,000 ng/kg bw /day. However, the German Federal Institute for Risk Assessment (BfR, 2023) has also presented its different views on the methodology of the EFSA re-evaluation, which suggests the use of this TDI value of 200 ng/kg bw/day as a basis for risk assessment. Nevertheless, the TDI of BPF or BPS remains to be established, in which case we assume they have the same TDI as BPA.

| Country | BPA | BPF | BPS | Reference |
| --- | --- | --- | --- | --- |
| EFSA TDI | 4,000 | 4,000 | 4,400 | EFSA (2015); Lin et al. (2022); Mok et al. (2021) |
| EFSA TDI | 0.2 | 0.2^a^ | 0.2 ^a^ | EFSA (2023) |
| BfR TDI | 200 | 200 ^a^ | 200 ^a^ | BfR (2023) |

^a^ The present study assume they have the same TDI as BPA.

*2.4 Measurement of renal function and other parameters in serum and urine*

The formula of related renal functions:

Estimated glomerular filtration rate (eGFR) _MDRD_ =

186 × (serum creatinine)^−1.154^ × (age)^−0.203^ × (0.742, if female) × (1.212, if black).

Estimated glomerular filtration rate (eGFR) _CKD-EPI_ =

141 × min (Scr/κ, 1)^α^ × max (Scr/κ, 1)^−1.209^ × 0.993^Age^ × 1.018 [if female] ×1.159 [if black], where Scr is serum creatinine, κ is 0.7 for female individuals and 0.9 for male individuals, α is −0.329 for female individuals and −0.411 for male individuals, min indicates the minimum of Scr/κor 1, and max indicates the maximum of Scr/κ or 1.

Estimated creatinine clearance rate (CCr) was calculated using the Cockcroft–Gault formula: $\text{CCr}\text{ }\text{=}\frac{\text{[(140 - age) × weight]}}{\text{(serum creatinine × 72) × (0.85, if female)}}$

Table S1. DI Estimation, Cumulative Risk Assessment of BPA and Substitutes, and Renal Function Calculation Formulas

| Item | Calculation Formulas |
| --- | --- |
| **BPA and its substitutes risk assessment** | |
| DI estimation | $DI of bisphenol [\frac{\left( \frac{\mu g}{kg} \right)}{day}]=\frac{UE \left( \frac{\mu g}{g} \right)\times CE (\frac{\frac{mg}{kg}}{day})}{F_{UE}\times BW \left( Kg \right)\times1000(\frac{mg}{g})}(Eq. 1)$ |
| Cumulative risk assessment | HQs: $\text{HQ}_{\mathrm{TDI}}\text{ }\text{=}\text{ }\frac{\mathrm{DI}}{\mathrm{TDI}}$  HI = ΣHQ |
| **Renal Function Calculation** | |
| Estimated glomerular filtration rate (eGFR) _MDRD_ | 186 × (serum creatinine)^−1.154^ × (age)^−0.203^ × (0.742, if female) × (1.212, if black). |
| Estimated glomerular filtration rate (eGFR) _CKD-EPI_ | 141 × min (Scr/κ, 1)^α^ × max (Scr/κ, 1)^−1.209^ × 0.993^Age^ × 1.018 [if female] ×1.159 [if black], where Scr is serum creatinine, κ is 0.7 for female individuals and 0.9 for male individuals, α is −0.329 for female individuals and −0.411 for male individuals, min indicates the minimum of Scr/κor 1, and max indicates the maximum of Scr/κ or 1. |
| Estimated creatinine clearance rate (CCr) | $\text{CCr}\text{ }\text{=}\frac{\text{[(140 - age) × weight]}}{\text{(serum creatinine × 72) × (0.85, if female)}}$ |

Table S2. Distribution of the study population according to the renal function (N = 366)

| Variables | Children/Adolescents   (<18 years, N = 95) | | |  | Adults  (≥18 years, n = 271) | | |
| --- | --- | --- | --- | --- | --- | --- | --- |
|  | abn. / N | abn. % | *p*^a^ |  | abn. / N | abn. % | *p*^a^ |
| BUN abn.^b^ |  |  |  |  |  |  |  |
| Total | 0/74 | 0 |  |  | 24/266 | 9.0 |  |
| Male | 0/45 | 0 | *-* |  | 14/126 | 11.1 | 0.259 |
| Female | 0/29 | 0 |  |  | 10/140 | 7.1 |  |
| Microalbumin abn.^c^ |  |  |  |  |  |  |  |
| Total | 9/95 | 9.47 |  |  | 28/271 | 10.3 |  |
| Male | 4/55 | 7.27 | 0.390 |  | 18/128 | 14.1 | 0.056 |
| Female | 5/40 | 12.5 |  |  | 10/143 | 7.0 |  |
| Protein abn.^d^ |  |  |  |  |  |  |  |
| Total | 9/95 | 9.5 |  |  | 18/271 | 6.6 |  |
| Male | 6/55 | 10.9 | 0.729 |  | 15/128 | 11.7 | 0.002 |
| Female | 3/40 | 7.5 |  |  | 3/143 | 2.1 |  |
| ACR abn.^e^ |  |  |  |  |  |  |  |
| Total | 8/95 | 8.4 |  |  | 22/271 | 8.1 |  |
| Male | 3/55 | 5.5 | 0.222 |  | 14/128 | 10.9 | 0.108 |
| Female | 5/40 | 12.5 |  |  | 8/143 | 5.6 |  |
| NAG/Creatinine abn.^f^ |  |  |  |  |  |  |  |
| Total | 4/92 | 4.4 |  |  | 108/269 | 40.2 |  |
| Male | 2/54 | 3.7 | 0.360 |  | 45/127 | 35.4 | 0.136 |
| Female | 2/38 | 5.3 |  |  | 63/142 | 44.4 |  |
| eGFR abn.^g^ |  |  |  |  |  |  |  |
| Total | 0/74 | 0 |  |  | 115/266 | 43.2 |  |
| Male | 0/45 | 0 | *-* |  | 63/126 | 50.0 | 0.035 |
| Female | 0/29 | 0 |  |  | 52/140 | 37.1 |  |
| Early chronic kidney disease ^h^ |  |  |  |  |  |  |  |
| Total | 0 | 0 |  |  | 101/266 | 38 |  |
| Male | 0 | 0 | *-* |  | 54/126 | 42.9 | 0.113 |
| Female | 0 | 0 |  |  | 47/140 | 33.6 |  |
| Type 2 DM ^i^ |  |  |  |  |  |  |  |
| Total | 0/95 | 0 |  |  | 30/271 | 11.1 |  |
| Male | 0/55 | 0 | *-* |  | 19/128 | 14.8 | 0.061 |
| Female | 0/40 | 0 |  |  | 11/143 | 7.7 |  |

BUN: blood urea nitrogen; ACR: albumin-to-creatinine ratio; NAG/CR: NAG-to-creatinine ratio; eGFR: Estimated glomerular filtration rate and based on CKD-MDRD equation; Type 2 DM: type 2 diabetes mellitus

^a^ Chi-squared test calculating the difference between sexes in each age group; ^b^ BUN > 20 mg/dL; ^c^ Microalbumin > 1.9 mg/dL; ^d^ Urine protein >14 mg/L; ^e^ ACR > 30 mg/g cre; ^f^ NAG/Creatinine > 4 IU/g; ^g^ eGFR < 90 mL/min/1.73 m^2^; ^h^ 60 ≤ eGFR < 90 mL/min/1.73 m^2^; ^i^ Fasting glucose ≥ 126 mg/dL or taking antidiabetic drugs.

Table S3. Adjusted regression coefficient and 95% CI for change in renal function index in relation to unit-increased in Log-BPs in Taiwanese adults (n=271)

| Analyte | Log-BUN | | | |  | Log-microalbumin | | | |  | Log-Protein | | | |  | Log-NAG | | | |  | Log-ACR | | | |
| --- | --- | --- | --- | --- | --- | --- | --- | --- | --- | --- | --- | --- | --- | --- | --- | --- | --- | --- | --- | --- | --- | --- | --- | --- |
|  | Beta | (95%CI) | | *P* |  | Beta | (95%CI) | | *P* |  | Beta | (95%CI) | | *P* |  | Beta | (95%CI) | | *P* |  | Beta | (95%CI) | | *P* |
| Model 1 ^a:^ concentrations (μg/L) | | | | |  |  |  |  |  |  |  |  |  |  |  |  |  |  |  |  |  |  |  |  |
| BPA | 0.34 | -0.16 | 0.83 | 0.183 |  | 2.00 | 0.09 | 3.91 | 0.040^*^ |  | 0.55 | -1.23 | 2.32 | 0.543 |  | -0.19 | -1.40 | 1.02 | 0.761 |  | 1.80 | -0.15 | 3.76 | 0.071 |
| BPF | 0.32 | -0.26 | 0.91 | 0.279 |  | 2.14 | -0.12 | 4.41 | 0.064 |  | 0.59 | -1.51 | 2.70 | 0.580 |  | -0.21 | -1.65 | 1.22 | 0.769 |  | 1.89 | -0.43 | 4.21 | 0.110 |
| BPS | 0.16 | 0.01 | 0.30 | 0.032^*^ |  | 0.69 | 0.13 | 1.24 | 0.016^*^ |  | 0.15 | -0.36 | 0.67 | 0.559 |  | -0.01 | -0.37 | 0.34 | 0.944 |  | 0.57 | 0.00 | 1.15 | 0.048^*^ |
| ΣBPs | -0.79 | -1.99 | 0.41 | 0.197 |  | -4.72 | -9.34 | -0.10 | 0.045^*^ |  | -1.46 | -5.76 | 2.83 | 0.503 |  | 0.42 | -2.51 | 3.35 | 0.777 |  | -4.12 | -8.86 | 0.61 | 0.088 |
| Model 2 ^b :^ daily intake (ng/kg/day) | | | | | | |  |  |  |  |  |  |  |  |  |  |  |  |  |  |  |  |  |  |
| BPA | -0.01 | -0.10 | 0.08 | 0.814 |  | -0.05 | -0.40 | 0.30 | 0.768 |  | -0.24 | -0.56 | 0.08 | 0.136 |  | -0.12 | -0.34 | 0.10 | 0.284 |  | 0.21 | -0.14 | 0.56 | 0.235 |
| BPF | -0.09 | -0.17 | -0.003 | 0.042^*^ |  | -0.29 | -0.61 | 0.04 | 0.086 |  | -0.33 | -0.62 | -0.03 | 0.031^*^ |  | -0.12 | -0.32 | 0.09 | 0.268 |  | -0.02 | -0.35 | 0.30 | 0.892 |
| BPS | 0.07 | -0.01 | 0.15 | 0.107 |  | 0.18 | -0.14 | 0.51 | 0.275 |  | -0.09 | -0.39 | 0.20 | 0.545 |  | -0.02 | -0.22 | 0.19 | 0.873 |  | 0.22 | -0.10 | 0.55 | 0.176 |
| Model 3 ^c^ | |  |  |  |  |  |  |  |  |  |  |  |  |  |  |  |  |  |  |  |  |  |  |  |
| HI | -0.04 | -0.08 | 0.01 | 0.137 |  | -0.18 | -0.37 | 0.01 | 0.057 |  | -0.66 | -0.83 | -0.49 | <0.001^***^ |  | -0.25 | -0.37 | -0.14 | <0.001^***^ |  | 0.39 | 0.21 | 0.58 | <0.001^***^ |

ΣBPs: the bisphenol weighted molar sum; To meet the normality assumption, the natural logarithm was used to transform the variables of renal function indexes; ^a^ Multiple regression analysis adjusted for urine creatinine, sex, age, BMI, having type II DM, and smoking habits; ^b^ Multiple regression analysis adjusted for sex, age, BMI, having type II DM, and smoking habits; ^c^ Based on TDI by EFSA (2015) and thresholds derived by Mok et al (2021), Lin et al (2022); ^*^: *p* < 0.05; ^**^: *p* < 0.01; ^***^: *p* < 0.001.

Table S4. Adjusted regression coefficient and 95% CI for change in renal function index in relation to unit-increased in Log-BPs in Taiwanese adults (n=271)

| Analyte | Log- NAG/Creatinine | | | |  | Log-eGFR ^d^ | | | |  | Log-eGFR^e^ | | | |
| --- | --- | --- | --- | --- | --- | --- | --- | --- | --- | --- | --- | --- | --- | --- |
|  | Beta | (95%CI) | | *P* |  | Beta | (95%CI) | | *P* |  | Beta | (95%CI) | | *P* |
| Model 1 ^a:^ concentrations (μg/L) | | | |  |  |  |  |  |  |  |  |  |  |  |
| BPA | -0.39 | -1.59 | 0.82 | 0.531 |  | -0.12 | -0.30 | 0.05 | 0.173 |  | -0.02 | -0.33 | 0.30 | 0.913 |
| BPF | -0.47 | -1.90 | 0.97 | 0.522 |  | -0.13 | -0.39 | 0.14 | 0.339 |  | -0.03 | -0.41 | 0.34 | 0.868 |
| BPS | -0.13 | -0.48 | 0.23 | 0.485 |  | -0.05 | -0.14 | 0.03 | 0.201 |  | -0.06 | -0.15 | 0.03 | 0.199 |
| ΣBPs | 1.02 | -1.90 | 3.95 | 0.492 |  | 0.27 | -0.23 | 0.77 | 0.289 |  | 0.10 | -0.66 | 0.87 | 0.793 |
| Model 2 ^b^ : daily intake (ng/kg/day) | | | |  |  |  |  |  |  |  |  |  |  |  |
| BPA | 0.14 | -0.07 | 0.36 | 0.195 |  | -0.03 | -0.09 | 0.02 | 0.214 |  | 0.03 | -0.03 | 0.08 | 0.339 |
| BPF | 0.15 | -0.06 | 0.35 | 0.161 |  | 0.05 | -0.01 | 0.11 | 0.092 |  | 0.02 | -0.03 | 0.07 | 0.424 |
| BPS | 0.03 | -0.18 | 0.23 | 0.793 |  | -0.001 | -0.06 | 0.06 | 0.982 |  | -0.05 | -0.10 | 0.003 | 0.067 |
| Model 3 ^c^ |  |  |  |  |  |  |  |  |  |  |  |  |  |  |
| HI | 0.32 | 0.21 | 0.44 | <.0001^***^ |  | 0.02 | -0.01 | 0.05 | 0.246 |  | 0.004 | -0.03 | 0.03 | 0.815 |

ΣBPs: the bisphenol weighted molar sum; eGFR: estimated glomerular filtration rate; To meet the normality assumption, the natural logarithm was used to transform the variables of renal function indexes; ^a^ Multiple regression analysis adjusted for urine creatinine, sex, age, BMI, having type II DM, and smoking habits; ^b^ Multiple regression analysis adjusted for sex, age, BMI, having type II DM, and smoking habits; ^c^ Based on TDI by EFSA (2015) and thresholds derived by Mok et al (2021), Lin et al (2022); ^d^ eGFR based on CKD- MDRD equation; ^e^ eGFR based on CKD- EPI equation.

Table S5. Schematic representation of the results obtained in the parameters related to renal function according to the bisphenols tertiles in adults.

| variables | BUN |  |  | Microalbumin |  |  | Protein |  |  | NAG |  |
| --- | --- | --- | --- | --- | --- | --- | --- | --- | --- | --- | --- |
|  | Median |  |  | Median |  |  | Median |  |  | Median |  |
|  | (Interquartile range) | P _trend_ ^a^ |  | (Interquartile range) | P _trend_ ^a^ |  | (Interquartile range) | P _trend_ ^a^ |  | (Interquartile range) | P _trend_ ^a^ |
| Concentrations (ug/L) | |  |  |  |  |  |  |  |  |  |  |
| BPA |  | 0.932 |  |  | 0.872 |  |  | 0.951 |  |  | 0.687 |
| <6.38 | 13.30 (9.65, 16.25) |  |  | 0.25 (0.25, 0.77) |  |  | 4.15 (2.90, 6.90) |  |  | 2.64 (1.81, 4.43) |  |
| 6.38-10.12 | 12.90 (10.40, 16.00) |  |  | 0.25 (0.25, 0.50) |  |  | 3.20 (2.10, 6.00) |  |  | 2.57 (1.54, 4.43) |  |
| >10.12 | 13.50 (10.65, 16.60) |  |  | 0.25 (0.25, 0.59) |  |  | 3.75 (2.20, 5.70) |  |  | 2.74 (1.64, 4.92) |  |
| BPF |  | 0.982 |  |  | 0.668 |  |  | 0.297 |  |  | 0.480 |
| <6.13 | 13.00 (9.80, 16.30) |  |  | 0.25 (0.25, 0.60) |  |  | 4.00 (2.60, 7.30) |  |  | 2.75 (1.81, 4.46) |  |
| 6.13-10.13 | 13.45 (10.30, 16.50) |  |  | 0.25 (0.25, 0.75) |  |  | 3.70 (2.30, 6.20) |  |  | 2.64 (1.58, 4.87) |  |
| >10.13 | 13.00 (10.50, 15.60) |  |  | 0.25 (0.25, 0.59) |  |  | 3.60 (2.10, 5.70) |  |  | 2.57 (1.59, 4.31) |  |
| BPS |  | 0.134 |  |  | 0.136 |  |  | 0.261 |  |  | 0.493 |
| <1.58 | 12.90 (9.60, 15.60) |  |  | 0.25 (0.25, 0.41) |  |  | 4.00 (2.40, 6.30) |  |  | 2.43 (1.68, 4.46) |  |
| 1.58-2.48 | 13.90 (10.00, 17.10) |  |  | 0.25 (0.25, 0.88) |  |  | 4.00 (2.80, 6.60) |  |  | 3.08 (1.92, 4.92) |  |
| >2.48 | 13.10 (10.75, 16.35) |  |  | 0.25 (0.25, 0.59) |  |  | 3.00 (1.90, 6.30) |  |  | 2.41 (1.38, 4.18) |  |
| ΣBPs |  | 0.864 |  |  | 0.979 |  |  | 0.541 |  |  | 0.951 |
| <0.07 | 13.00 (9.80, 16.30) |  |  | 0.25 (0.25, 0.64) |  |  | 4.55 (2.80, 7.20) |  |  | 2.64 (1.85, 4.46) |  |
| 0.07-0.10 | 13.60 (9.90, 16.40) |  |  | 0.25 (0.25, 0.60) |  |  | 3.30 (2.20, 5.80) |  |  | 2.52 (1.53, 4.61) |  |
| >0.10 | 13.10 (10.75, 16.40) |  |  | 0.25 (0.25, 0.64) |  |  | 3.60 (2.10, 6.80) |  |  | 2.57 (1.59, 4.61) |  |
| Daily intake (ng/kg/day) | |  |  |  |  |  |  |  |  |  |  |
| BPA DI |  | 0.417 |  |  | 0.378 |  |  | 0.011^**^ |  |  | 0.001^***^ |
| <1.64 | 13.55 (9.75, 16.35) |  |  | 0.44 (0.44, 1.16) |  |  | 6.00 (3.90, 8.90) |  |  | 3.23 (2.13, 4.87) |  |
| 1.64-3.61 | 13.10 (10.40, 16.10) |  |  | 0.25 (0.25, 0.60) |  |  | 4.00 (2.40, 5.80) |  |  | 2.60 (1.61, 5.07) |  |
| >3.61 | 13.10 (10.65, 16.35) |  |  | 0.25 (0.25, 0.25) |  |  | 2.60 (1.60, 3.70) |  |  | 2.03 (1.48, 3.84) |  |
| BPF DI |  | 0.321 |  |  | 0.354 |  |  | 0.014^**^ |  |  | 0.001^***^ |
| <1.61 | 13.40 (9.80, 16.20) |  |  | 0.29 (0.29, 1.06) |  |  | 5.85 (3.80, 8.80) |  |  | 3.23 (2.16, 5.15) |  |
| 1.61-3.46 | 13.90 (11.00, 16.30) |  |  | 0.25 (0.25, 0.60) |  |  | 3.70 (2.70, 5.90) |  |  | 2.57 (1.59, 4.93) |  |
| >3.46 | 12.55 (9.65, 16.05) |  |  | 0.25 (0.25, 0.28) |  |  | 2.60 (1.60, 4.00) |  |  | 2.00 (1.36, 4.00) |  |
| BPS DI |  | 0.623 |  |  | 0.963 |  |  | 0.101 |  |  | 0.001^***^ |
| <0.41 | 13.10 (9.90, 15.00) |  |  | 0.25 (0.25, 0.88) |  |  | 5.80 (4.10, 8.80) |  |  | 3.83 (2.19, 5.64) |  |
| 0.41-0.81 | 14.10 (11.00, 18.00) |  |  | 0.25 (0.25, 0.53) |  |  | 3.40 (2.60, 5.70) |  |  | 2.19 (1.42, 3.89) |  |
| >0.81 | 12.90 (9.90, 15.85) |  |  | 0.25 (0.25, 0.29) |  |  | 2.60 (1.60, 4.00) |  |  | 2.33 (1.53, 4.15) |  |

ΣBPs: the bisphenol weighted molar sum; NAG: N-acetyl-β-D-glucosaminidase; ^a^ P for trend values calculated by tertiles using a General Linear Model (GLM); ^*^: *p* < 0.05; ^**^: *p* < 0.01; ^***^: *p* < 0.001.

Table S6. Schematic representation of the results obtained in the parameters related to renal function according to the bisphenols tertiles in adults.

| variables | ACR |  |  | NAG/Creatinine |  |  | eGFR ^b^ |  |  | eGFR ^c^ |  |  |
| --- | --- | --- | --- | --- | --- | --- | --- | --- | --- | --- | --- | --- |
|  | Median |  |  | Median |  |  | Median |  |  | Median |  |  |
|  | (Interquartile range) | P _trend_ ^a^ |  | (Interquartile range) | P _trend_ ^a^ |  | (Interquartile range) | P _trend_ ^a^ |  | (Interquartile range) | P _trend_ ^a^ |  |
| Concentrations (ug/L) | | |  |  |  |  |  |  |  |  |  |  |
| BPA |  | 0.859 |  |  | 0.997 |  |  | 0.685 |  |  | 0.935 |  |
| <5.48 | 4.48 (2.79, 9.57) |  |  | 3.22 (1.96, 5.63) |  |  | 96.81 (82.85, 110.81) |  |  | 95.14 (82.48, 110.63) |  |  |
| 5.48-9.34 | 4.39 (2.69, 7.35) |  |  | 3.13 (1.81, 5.88) |  |  | 96.46 (83.05, 110.71) |  |  | 94.76 (83.39, 110.50) |  |  |
| >9.34 | 4.72 (2.92, 7.14) |  |  | 3.76 (2.17, 6.60) |  |  | 92.51 (82.04, 109.25) |  |  | 94.15 (82.72, 109.98) |  |  |
| BPF |  | 0.753 |  |  | 0.515 |  |  | 0.728 |  |  | 0.811 |  |
| <5.99 | 3.88 (2.62, 7.31) |  |  | 3.33 (1.96, 5.76) |  |  | 95.16 (83.62, 106.47) |  |  | 94.44 (83.88, 106.64) |  |  |
| 5.99-9.91 | 4.55 (2.75, 8.06) |  |  | 3.03 (1.83, 5.39) |  |  | 94.33 (82.18, 110.06) |  |  | 94.13 (82.55, 110.80) |  |  |
| >9.91 | 4.76 (3.01, 7.61) |  |  | 3.61 (2.17, 7.25) |  |  | 92.59 (82.15, 115.24) |  |  | 95.34 (82.76, 115.87) |  |  |
| BPS |  | 0.495 |  |  | 0.551 |  |  | 0.814 |  |  | 0.983 |  |
| <1.52 | 4.27 (2.58, 7.31) |  |  | 3.39 (2.00, 6.02) |  |  | 97.74 (82.14, 109.18) |  |  | 94.44 (82.27, 109.70) |  |  |
| 1.52-2.48 | 4.81 (3.09, 7.47) |  |  | 3.60 (2.24, 5.81) |  |  | 93.07 (83.39, 115.24) |  |  | 94.11 (83.81, 115.67) |  |  |
| >2.48 | 4.39 (2.78, 7.64) |  |  | 3.05 (1.79, 6.13) |  |  | 93.33 (82.54, 109.35) |  |  | 95.18 (82.39, 109.56) |  |  |
| ΣBPs |  | 0.933 |  |  | 0.396 |  |  | 0.717 |  |  | 0.756 |  |
| <0.07 | 3.91 (2.48, 7.31) |  |  | 3.22 (1.83, 5.76) |  |  | 99.02 (83.62, 109.93) |  |  | 96.25 (83.91, 109.91) |  |  |
| 0.07-0.10 | 4.55 (2.89, 7.61) |  |  | 2.94 (2.00, 5.66) |  |  | 94.30 (82.95, 110.71) |  |  | 94.16 (82.55, 110.24) |  |  |
| >0.10 | 4.89 (3.01, 7.44) |  |  | 3.76 (2.24, 6.74) |  |  | 90.06 (81.72, 110.32) |  |  | 93.77 (81.89, 110.02) |  |  |
| Daily intake (ng/kg/day) | | |  |  |  |  |  |  |  |  |  |  |
| BPA |  | 0.428 |  |  | <0.001^***^ |  |  | 0.460 |  | 96.45 (83.52, 115.31) | 0.274 |  |
| <1.64 | 3.52 (2.00, 7.47) |  |  | 2.59 (1.58, 3.74) |  |  | 96.82 (83.55, 115.69) |  |  | 94.03 (82.91, 109.24) |  |  |
| 1.64-3.61 | 3.91 (2.60, 5.95) |  |  | 3.22 (2.00, 5.81) |  |  | 94.58 (82.14, 109.03) |  |  | 93.47 (82.43, 110.44) |  |  |
| >3.61 | 5.43 (3.74, 8.06) |  |  | 4.54 (2.91, 8.20) |  |  | 92.44 (82.16, 110.32) |  |  |  |  |  |
| BPF |  | 0.456 |  |  | <0.001^***^ |  |  | 0.800 |  |  | 0.913 |  |
| <1.61 | 3.34 (2.00, 6.25) |  |  | 2.71 (1.74, 4.21) |  |  | 95.07 (84.47, 109.18) |  |  | 94.37 (84.34, 109.91) |  |  |
| 1.61-3.46 | 4.55 (2.94, 7.44) |  |  | 3.27 (1.98, 6.28) |  |  | 94.67 (80.12, 107.30) |  |  | 95.29 (80.55, 107.25) |  |  |
| >3.46 | 5.43 (3.73, 8.93) |  |  | 4.15 (2.69, 8.63) |  |  | 92.52 (82.61, 115.04) |  |  | 94.65 (82.43, 115.02) |  |  |
| BPS |  | 0.166 |  |  | <0.001^***^ |  |  | 0.391 |  |  | 0.304 |  |
| <0.41 | 3.49 (2.00, 5.95) |  |  | 2.81 (1.66, 4.53) |  |  | 95.16 (85.42, 109.93) |  |  | 95.56 (85.49, 109.87) |  |  |
| 0.41-0.81 | 3.98 (2.75, 7.31) |  |  | 3.11 (1.83, 4.34) |  |  | 98.09 (81.51, 114.37) |  |  | 94.11 (81.98, 114.55) |  |  |
| >0.81 | 5.43 (3.43, 10.87) |  |  | 4.56 (2.59, 8.56) |  |  | 91.27 (82.61, 109.02) |  |  | 94.03 (82.43, 109.39) |  |  |

ΣBPs: the bisphenol weighted molar sum; ACR: microalbumin-to-creatinine ratio; eGFR: estimated glomerular filtration rate; ^a^ P for trend values calculated by tertiles using a General Linear Model (GLM); ^b^ eGFR based on CKD- MDRD equation; ^c^ eGFR based on CKD- EPI equation; ^*^: *p* < 0.05; ^**^: *p* < 0.01; ^***^: *p* < 0.001.

Table S7. Association between BPs levels and the risk of higher renal function indexes in adults (n=271).

| Analyte | eGFR^a^ | | |  | Early chronic kidney disease ^b^ | | |  | Early chronic kidney disease ^c^ | | |
| --- | --- | --- | --- | --- | --- | --- | --- | --- | --- | --- | --- |
|  | Case/ N (%) | AOR (95%CI) | *P* |  | Case/ N (%) | AOR (95%CI) | *P* |  | Case/ N (%) | AOR (95%CI) | *P* |
| *Model 1* ^d^ |  |  |  |  |  |  |  |  |  |  |  |
| BPA |  |  |  |  |  |  |  |  |  |  |  |
| <6.38 | 35/88 (39.8) | 1 | - |  | 27/88 (30.7) | 1 | - |  | 28/88 (31.8) | 1 | - |
| 6.38-10.12 | 35/90 (38.9) | 1.69 (0.65, 4.42) | 0.282 |  | 30/90 (33.3) | 1.85 (0.82, 4.18) | 0.138 |  | 40/90 (44.4) | 2.50 (1.19, 5.24) | 0.015 |
| >10.12 | 35/88 (39.8) | 0.85 (0.28, 2.56) | 0.778 |  | 29/88 (33) | 1.37 (0.54, 3.47) | 0.504 |  | 45/88 (51.1) | 2.55 (1.09, 5.95) | 0.031 |
| BPF |  |  |  |  |  |  |  |  |  |  |  |
| <6.13 | 33/87 (37.9) | 1 | - |  | 28/87 (32.2) | 1 | - |  | 33/87 (37.9) | 1 | - |
| 6.13-10.13 | 37/90 (41.1) | 1.21 (0.47, 3.12) | 0.692 |  | 29/90 (32.2) | 1.06 (0.47, 2.39) | 0.892 |  | 37/90 (41.1) | 0.93 (0.45, 1.93) | 0.846 |
| >10.13 | 35/89 (39.3) | 1.06 (0.36, 3.13) | 0.918 |  | 29/89 (32.6) | 1.12 (0.44, 2.81) | 0.817 |  | 43/89 (48.3) | 1.16 (0.52, 2.63) | 0.715 |
| BPS |  |  |  |  |  |  |  |  |  |  |  |
| <1.58 | 34/88 (38.2) | 1 | - |  | 31/88 (34.8) | 1 | - |  | 34/89 (38.2) | 1 | - |
| 1.58-2.48 | 35/89 (39.3) | 1.03 (0.40, 2.66) | 0.951 |  | 27/89 (30.3) | 0.64 (0.28, 1.45) | 0.287 |  | 36/89 (40.5) | 0.79 (0.38, 1.64) | 0.526 |
| >2.48 | 36/88 (40.9) | 1.92 (0.64, 5.71) | 0.242 |  | 28/88 (31.8) | 0.78 (0.31, 1.95) | 0.593 |  | 43/88 (48.9) | 1.14 (0.50, 2.62) | 0.754 |
| *Model 2* ^e^ |  |  |  |  |  |  |  |  |  |  |  |
| BPA DI |  |  |  |  |  |  |  |  |  |  |  |
| <1.64 | 33/88 (37.5) | 1 | - |  | 27/88 (30.7) | 1 | - |  | 33/88 (37.5) | 1 | - |
| 1.64-3.61 | 34/90 (37.8) | 1.08 (0.37, 3.12) | 0.894 |  | 26/90 (28.9) | 1.02 (0.42, 2.50) | 0.964 |  | 40/90 (44.4) | 1.37 (0.62, 3.02) | 0.441 |
| >3.61 | 38/88 (43.2) | 0.49 (0.11, 2.10) | 0.336 |  | 33/88 (37.5) | 1.01 (0.29, 3.47) | 0.988 |  | 40/88 (45.5) | 0.91 (0.29, 2.79) | 0.862 |
| BPF DI |  |  |  |  |  |  |  |  |  |  |  |
| <1.61 | 34/89 (38.2) | 1 | - |  | 28/89 (31.5) | 1 | - |  | 35/89 (39.3) | 1 | - |
| 1.61-3.46 | 35/89 (39.3) | 0.89 (0.31, 2.58) | 0.831 |  | 27/89 (30.3) | 0.94 (0.39, 2.26) | 0.887 |  | 39/89 (43.8) | 1.10 (0.51, 2.40) | 0.803 |
| >3.46 | 36/88 (40.9) | 0.73 (0.17, 3.02) | 0.658 |  | 31/88 (35.2) | 0.96 (0.28, 3.29) | 0.951 |  | 39/88 (44.3) | 0.76 (0.25, 2.30) | 0.625 |
| BPS DI |  |  |  |  |  |  |  |  |  |  |  |
| <0.41 | 30/89 (33.7) |  |  |  | 27/89 (30.3) | 1 | - |  | 35/89 (39.3) | 1 | - |
| 0.41-0.81 | 38/89 (42.7) | 1.73 (0.62, 4.85) | 0.296 |  | 28/89 (31.5) | 0.84 (0.35, 1.98) | 0.682 |  | 35/89 (39.3) | 0.89 (0.41, 1.95) | 0.775 |
| >0.81 | 37/88 (42.1) | 4.06 (0.97, 17.07) | 0.056^†^ |  | 31/88 (35.2) | 1.33 (0.41, 4.32) | 0.631 |  | 43/88 (48.9) | 2.29 (0.79, 6.62) | 0.127 |
| *Model 3* ^f^ |  |  |  |  |  |  |  |  |  |  |  |
| HI ^e^ |  |  |  |  |  |  |  |  |  |  |  |
| <0.001 | 34/89 (38.2) | 1 | - |  | 28/89 (31.5) | 1 | - |  | 35/89 (39.3) | 1 | - |
| 0.001-0.002 | 32/89 (36) | 1.01 (0.41, 2.47) | 0.982 |  | 25/89 (28.1) | 0.87 (0.40, 1.87) | 0.719 |  | 36/89 (40.5) | 1.17 (0.60, 2.26) | 0.652 |
| >0002 | 39/88 (44.3) | 1.19 (0.50, 2.82) | 0.687 |  | 33/88 (37.5) | 1.24 (0.59, 2.62) | 0.577 |  | 42/88 (47.7) | 1.44 (0.74, 2.81) | 0.285 |

eGFR: Estimated glomerular filtration rate; AOR: Adjusted Odds ratio; *p*: p-value; ^a^ eGFR < 90 mL/min/1.73 m^2^ based on CKD-EPI equation; ^b^ 60 ≤ eGFR < 90 mL/min/1.73 m^2^, and eGFR based on CKD-EPI equation; ^c^ 60 ≤ eGFR < 90 mL/min/1.73 m^2^ or urine protein >14 mg/L, and eGFR based on CKD-MDRD equation; ^c^ Adjustment of age, sex, type 2 DM, urine creatinine and BMI; ^d^ Adjustment of age, sex, type 2 DM, and BMI ^†^ : *p* < 0.01;^*^: *p* < 0.05; ^**^: *p* < 0.01; ^***^: *p* < 0.001.

Table S8. Association between BPs HI and the risk of higher renal function indexes in adults (n=271).

| HI value | NAG/Creatinine ^a^ | | |
| --- | --- | --- | --- |
| Model 1^b^ | Case/ N (%) | AOR (95%CI) | *p* |
| <0.001 | 21/90 (23.3) | 1 | - |
| 0.001-0.002 | 35/90 (38.9) | 2.18 (1.19, 4.34) | 0.027^*^ |
| >0.002 | 52/89 (58.4) | 4.27 (1.19, 8.51) | <0.001^***^ |
| Model 2^c^ |  |  |  |
| <18.68 | 21/90 (23.3) | 1.00 | - |
| 18.68-40.23 | 35/90 (38.9) | 2.18 (1.10, 4.34) | 0.027^*^ |
| >40.23 | 52/89 (58.4) | 4.27 (2.14, 8.51) | <0.001^***^ |
| Model 3^d^ |  |  |  |
| <0.02 | 25/90 (27.8) | 1.00 | - |
| 0.02-0.05 | 32/91 (35.2) | 1.41 (0.73, 2.76) | 0.309 |
| >0.05 | 51/88 (58) | 3.33 (1.70, 6.52) | <0.001^***^ |

NAG/creatinine: NAG-to-creatinine ratio; AOR: Adjusted Odds ratio; *p*: p-value; HI is the cumulative summation of HQs for each compound; ^a^ NAG/Creatinine > 4 IU/g; ^b^ Model 1: based on EFSA (2015) TDI of BPA (4,000 ng/kg/day), TDI of 4,000 (ng/kg bw /day) for BPF (Lin et al., 2022); TDI of 4,400 (ng/kg bw /day) for BPS; ^c^ Model 2: based on EFSA (2023) TDI of BPA (0.2 ng/kg/day) and assume the BPA TDI equal to BPF and BPS; ^d^ Model 3: based on BfR (2023) TDI (200 ng/kg/day) and assume the BPA TDI equal to BPF and BPS; Adjustment of age, sex, type 2 DM and BMI in all models; ^*^: *p* < 0.05; ^**^: *p* < 0.01; ^***^: *p* < 0.001.


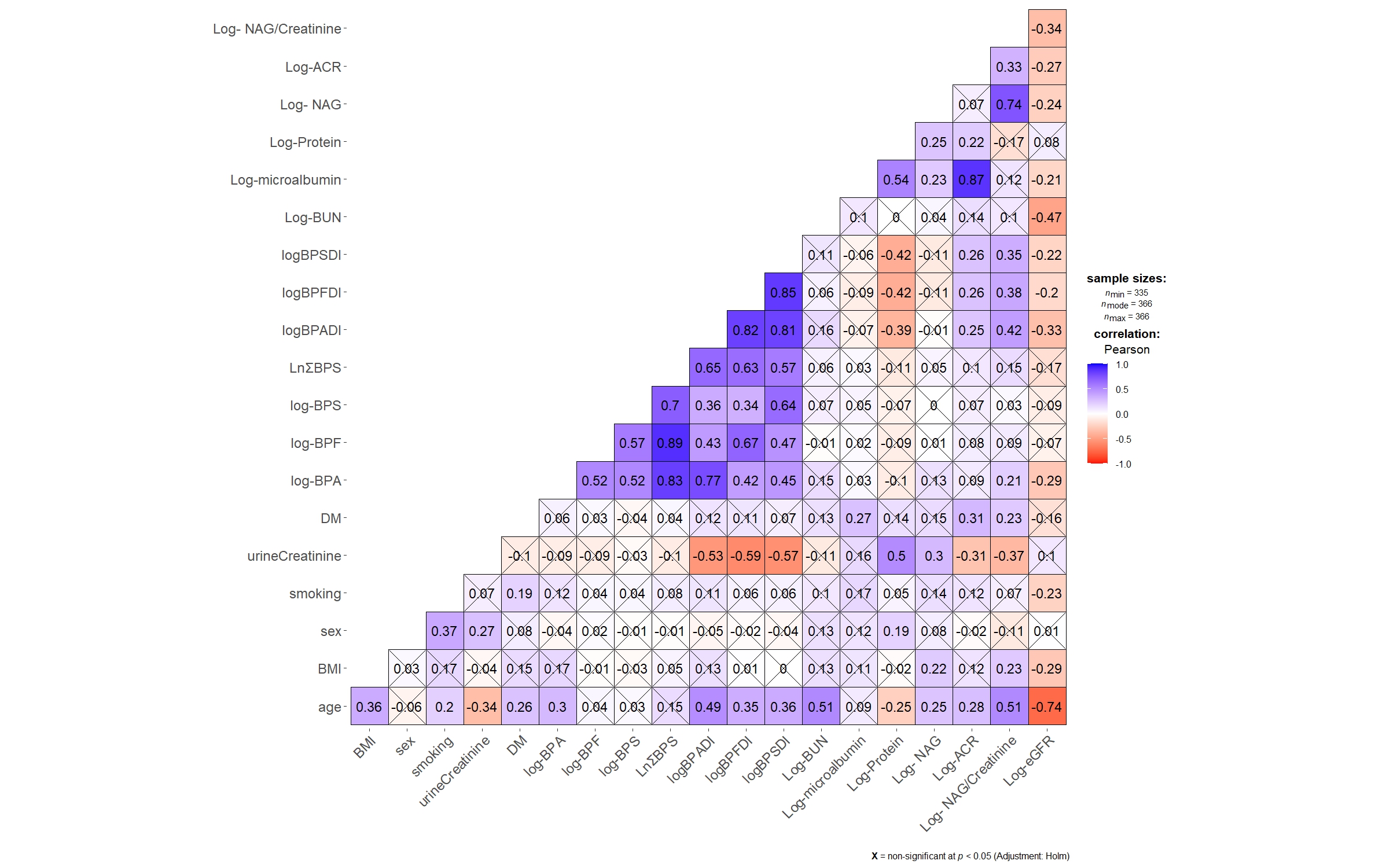


Fig. S1. Correlations among bisphenols and renal function in RPL groups in Taiwanese (n=366). Cross mark means correlation is not significant (p-value ≥ 0.05)


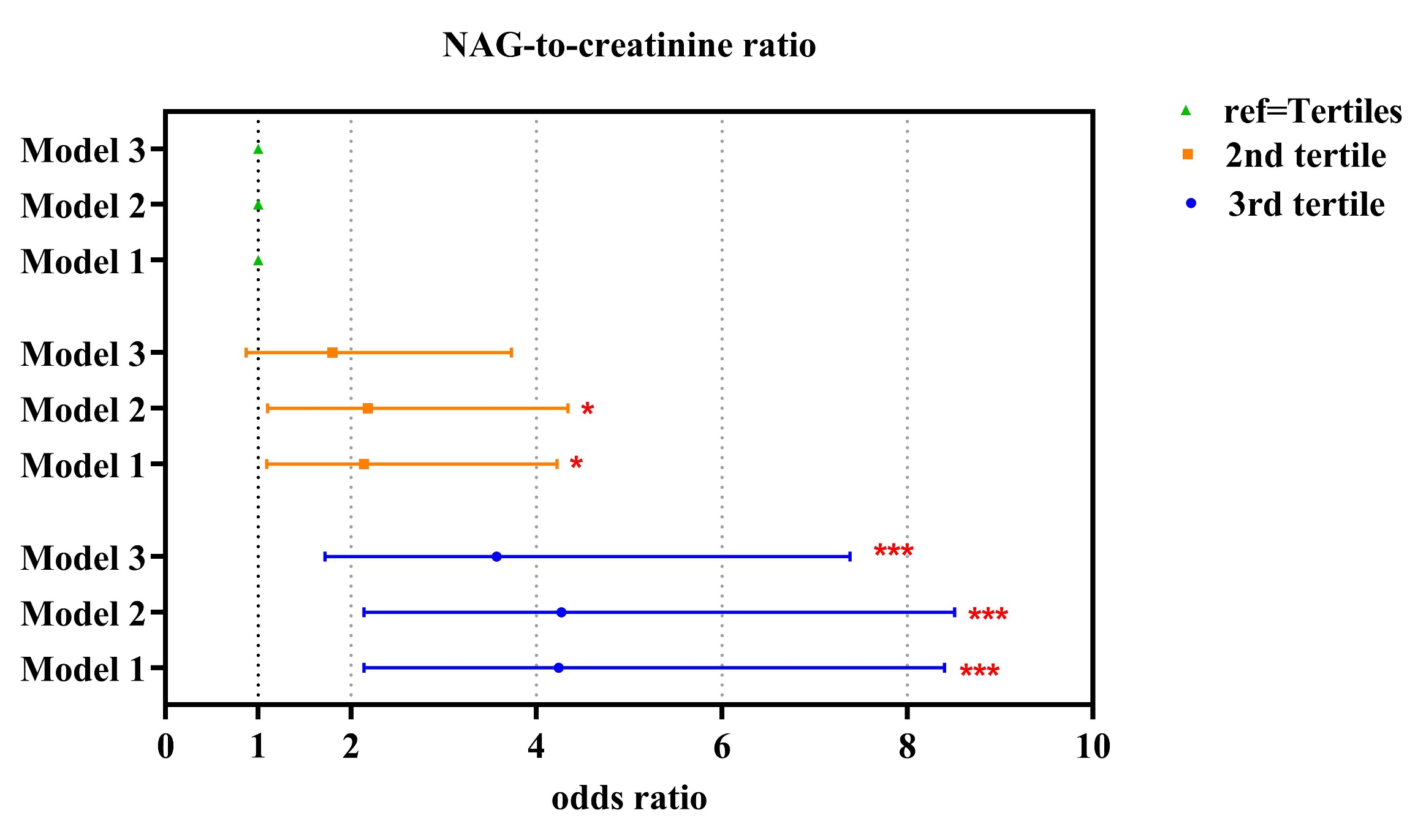


Fig. S2. Association between the HI _kidney toxicity effects_ and renal function index of NAG-to-creatinine ratio. Model 1: Age greater than 18 y, no excluding participants with type 2 DM, and adjusted for BMI, sex and age; Model 2: Age greater than 18 y and adjusted for BMI, sex, age and participants with type 2 DM; Model 3: Age greater than 18 y, excluding participants with type 2 DM, and adjusted for BMI, sex and age. ^*^: *p* < 0.05; ^**^: *p* < 0.01; ^***^: *p* < 0.001.

| 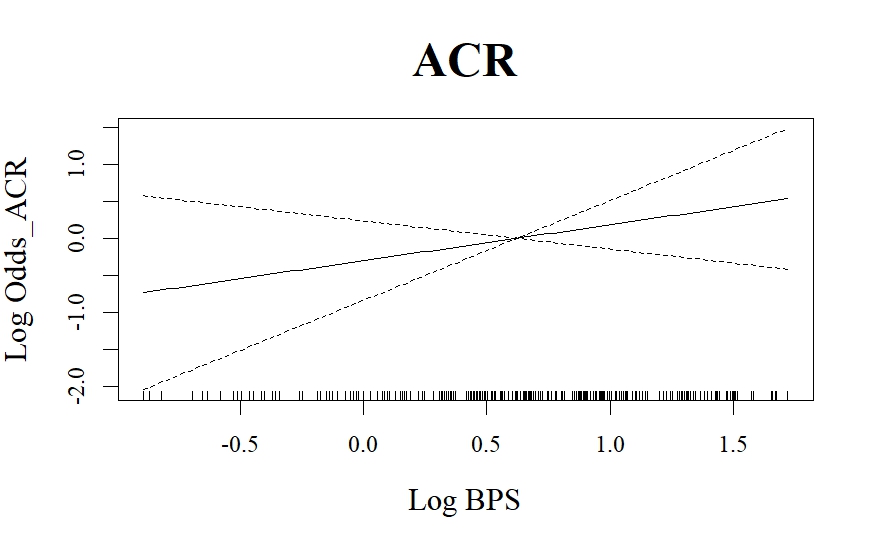 | 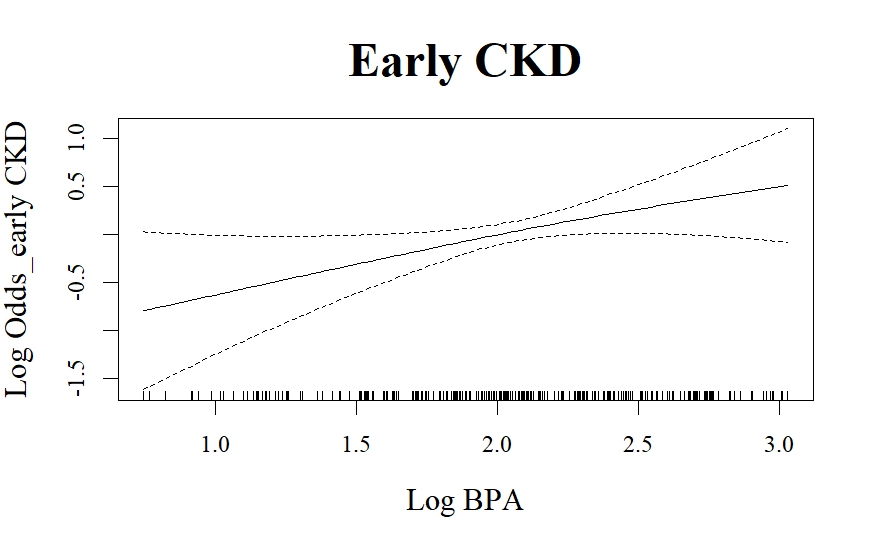 |
| --- | --- |
| 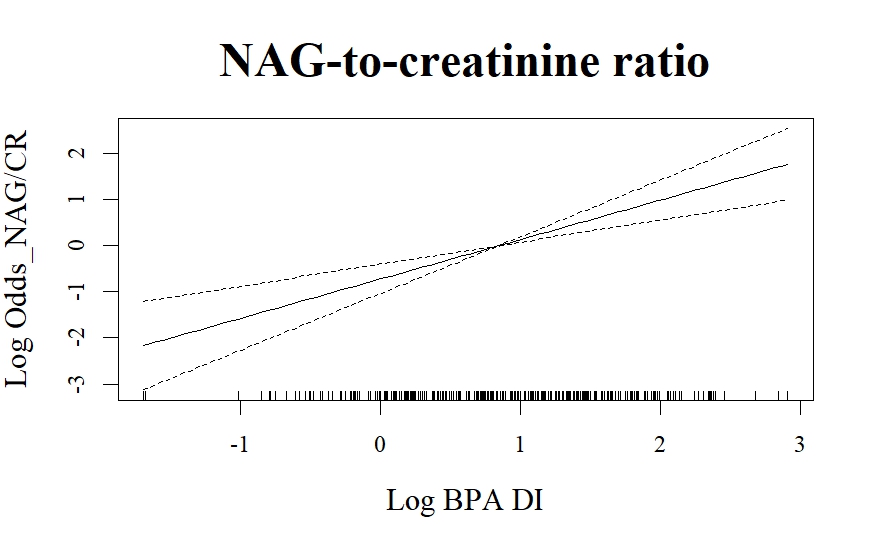 | 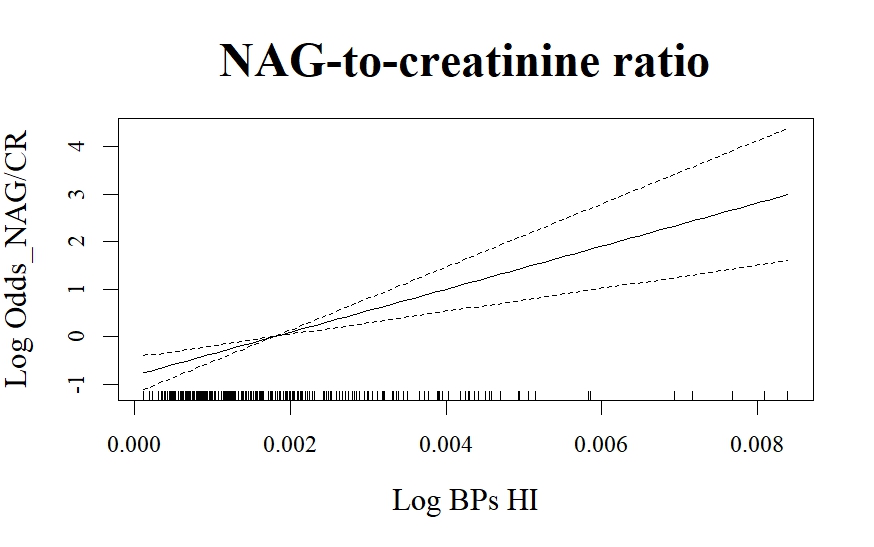 |

Fig. S3. Relationship between concentrations of bisphenols levels and renal function index in the penalized regression splines; both the fitted (solid line) and 95% confidence interval (dotted lines) lines are presented (P_smooth_<0.05).

**References:**

EFSA (European Food Safety Authority). (2015). Scientific opinion on the risks to public health related to the presence of bisphenol a (BPA) in foodstuffs. EFSA J. 13(1): 3978. doi: 10.2903/j.efsa.2015.3978

Mok, S., Jeong, Y., Park, M., Kim, S., Lee, I., Park, J., Kim, S., Choi, K., & Moon, H. B. (2021). Exposure to phthalates and bisphenol analogues among childbearing-aged women in Korea: Influencing factors and potential health risks. Chemosphere. 264(Pt1):128425. doi: 10.1016/j.chemosphere.2020.128425

Lin, N., Ma, D., Liu, Z., Wang, X., Ma, L. (2022). Migration of bisphenol A and its related compounds in canned seafood and dietary exposure estimation. Food Quality and Safety. 6:fyac006. https://doi.org/10.1093/fqsafe/fyac006
